# AZP-531, an unacylated ghrelin analog, improves food-related behavior in patients with Prader-Willi syndrome: A randomized placebo-controlled trial

**DOI:** 10.1371/journal.pone.0190849

**Published:** 2018-01-10

**Authors:** Soraya Allas, Assumpta Caixàs, Christine Poitou, Muriel Coupaye, Denise Thuilleaux, Françoise Lorenzini, Gwenaëlle Diene, Antonino Crinò, Frédéric Illouz, Graziano Grugni, Diane Potvin, Sarah Bocchini, Thomas Delale, Thierry Abribat, Maithé Tauber

**Affiliations:** 1 Alizé Pharma, Ecully, France; 2 Department of Endocrinology and Nutrition, Parc Taulí Hospital Universitari, Institut d’Investigació in Innovació Parc Taulí I3PT, Universitat Autònoma de Barcelona, Sabadell, Spain; 3 Department of Nutrition, Institute of Cardiometabolism and Nutrition, Pitié-Salpêtrière Hospital, Assistance Publique-Hôpitaux de Paris, Paris, France; 4 Department of Rare Diseases, Marin Hospital, Hendaye, France; 5 Rangueil Hospital, Toulouse, France; 6 Department of Endocrinology, Bone Diseases, Genetics, and Gynaecology, Children's Hospital, Toulouse, France; 7 Autoimmune Endocrine Diseases Unit, Bambino Gesù, Research Hospital, Palidoro, Rome, Italy; 8 Department of Endocrinology, Diabetes and Nutrition, Angers’ Hospital, Angers, France; 9 Division of Auxology, Istituto Auxologico Italiano Piancavallo, Verbania, Italy; 10 Excelsus Statistics, Montréal, Quebec, Canada; 11 INSERM U1043, Centre de Physiopathologie de Toulouse-Purpan, Université Paul Sabatier, Toulouse, France; Vanderbilt University, UNITED STATES

## Abstract

**Context and objective:**

Prader-Willi syndrome (PWS) is characterized by early-onset hyperphagia and increased circulating levels of the orexigenic Acylated Ghrelin (AG) hormone with a relative deficit of Unacylated Ghrelin (UAG). AZP-531, a first-in-class UAG analog, was shown to inhibit the orexigenic effect of AG in animals, to improve glycemic control and decrease body weight in humans. We aimed to investigate the safety and efficacy of AZP-531 in patients with PWS for whom no approved treatment for hyperphagia is currently available.

**Methods and design:**

Multi-center, randomized, double-blind, placebo-controlled trial. Forty-seven patients with genetically confirmed PWS and evidence of hyperphagia received daily subcutaneous injections of AZP-531 (3 and 4 mg for 50–70 kg and >70 kg body weight, respectively) or matching placebo for 14 days. Assessments included adverse events, vital signs, safety laboratory tests, the Hyperphagia Questionnaire (HQ), patient-reported appetite, body composition and glycemic measures.

**Results:**

AZP-531 was well tolerated. There was a significant improvement with AZP-531 versus placebo in the mean total score, the 9-item score and the severity domain score of the HQ (p < .05). The highest reduction in the total and 9-item scores was observed in AZP-531 subjects with the highest hyperphagia score at baseline. Findings were supported by a reduction in appetite scores observed with AZP-531 only. Body weight did not change in both groups while a significant reduction in waist circumference and fat mass was observed only with AZP-531. AZP-531 significantly decreased post-prandial glucose levels in a baseline glucose dependent fashion.

**Conclusions:**

AZP-531 may constitute a new treatment strategy to improve hyperphagia and metabolic issues in patients with PWS. These findings support further investigation in longer-term clinical trials.

## Introduction

Prader-Willi Syndrome is a rare genetic disorder that results from lack of expression of paternally inherited imprinted genes in chromosomal region 15q11.2-q13 caused either by paternal deletion (65–75%), maternal uniparental disomy (20–30%) or imprinting defects [1]. Hyperphagia is the central and constant feature of the syndrome starting after the first 3 years of life and is associated with abnormal and extreme behaviors toward food [2, 3]. Hyperphagia and food-related behaviors dramatically impair socialization and occupational performance and substantially deteriorate quality of life of patients and caregivers [4, 5] and is responsible for significant morbidity and mortality [6]. There is no approved pharmacological treatment for hyperphagia.

Ghrelin is a 28-amino-acid gut-derived hormone that circulates in 2 forms: 1) Acylated ghrelin (AG) that binds the Growth Hormone Secretagogue Receptor (GHSR) and has orexigenic, obesogenic and diabetogenic properties [7–9] and 2) Unacylated ghrelin (UAG) that acts through GHSR independent pathways and was shown to inhibit these effects [10–13]. Both molecules share protective effects from oxidative stress and inflammation on various cell types including ß-cells, cardiomyocytes, skeletal muscle cells and endothelial progenitor cells that have been shown in vitro and in vivo [14–18]. Many reports have documented that fasting and post-prandial circulating levels of total ghrelin are elevated in PWS at all ages, as compared to lean and obese subjects [19–24]. A large recent study that included 138 children and adults with PWS has shown that this is due to high levels of AG and relatively low levels of UAG that are observed when hyperphagia and obesity develop [25]. In PWS, ghrelin levels have been shown to positively correlate to ratings of hunger [22] and because of the role of AG in stimulating appetite, elevated AG levels in PWS are hypothesized to contribute to hyperphagia.

Antagonizing or blocking the effects of AG on food intake, body weight and glucose metabolism has emerged in the past decade as an attractive pharmaco-therapeutic target. Antagonists and inverse agonists of the AG receptor GHSR, as well as AG-blocking agents have been designed to this end, but mixed results have been obtained so far in animal models while clinical efficacy data are lacking ([26, 27]). On the other hand, inhibitors of the Ghrelin O-Acyltransferase (GOAT) enzyme responsible for acylation are still at an early stage of investigation and no efficacy data is available [27]. UAG is not an antagonist of GHSR and acts as a functional inhibitor of AG, which has appeared as a valuable approach particularly as it may benefit clinical conditions associated with elevated circulating AG levels and a relative deficit in circulating UAG levels such as PWS. As opposed to approaches targeting GHSR or AG, this approach may in addition preserve protective effects from oxidative stress and inflammation on tissues, which is of particular relevance in metabolic disorders.

AZP-531 is a cyclic 8 amino-acid analog of UAG with improved plasma stability and pharmacokinetics [28] and reproduces the pharmacological effects of UAG [11, 29]. In humans, AZP-531 has a mean half-life of 3 hours and is suitable for once-daily dosing and has been shown to improve glucose control and decrease weight in Phase 1 clinical trials [30]. As a first investigation in patients with PWS, the present study has been designed as a 2-week proof-of-concept study to assess the safety and efficacy of AZP-531 administration in improving food-related behaviors. Additional assessments included body composition and glycemic measures.

## Materials and methods

### Trial design and overview

This study was a randomized, double-blind, placebo-controlled trial conducted between January 19, 2015 and January 7, 2016 at 7 clinical research and university-affiliated sites with strong medical expertise in PWS located in France, Spain, and Italy (EudraCT: 2014-001670-34).

The protocol for this trial and supporting CONSORT checklist are available as supporting information; see S1 CONSORT Checklist and S1 Study Protocol.

The trial was conducted in strict accordance with the principles expressed in the Declaration of Helsinki and Good Clinical Practice The study protocol was approved by the respective national Regulatory Bodies and by the following ethics committees:

- For Spain: Comité Ético de Investigación Clínica de la Corporació Sanitària Parc Taulí de Sabadell (Favorable opinion received on November 04th 2014).
- For France: Comité de Protection des Personnes Sud-Ouest et Outre-Mer II (Favorable opinion received on November 07th 2014);
- For Italy: Comitato Etico dell’Ospedale Pediatrico Bambino Gesù (Favorable opinion received on July 17th 2015)

Eligible patients were randomly assigned in a ratio of 1:1 to receive daily subcutaneous (sc) injections of either AZP-531 (3 and 4 mg for 50–70 kg and >70 kg body weight, respectively) or matching placebo, 30 minutes prior to breakfast for 14 days. Dose selection was based on data from previous clinical studies in obese subjects and patients with Type 2 diabetes showing that daily administration of AZP-531 at doses of 30 μg/kg and 60 μg/kg for 2 weeks were well tolerated and were associated with pharmacodynamic effects [30]. The study drugs were provided as lyophilized powder for reconstitution in 0.9% sodium chloride for injection and were of identical appearance. Randomization was stratified based on genetic subtype (deletion, non-deletion) and was performed using an Interactive Web Response System according to a computer-generated randomization schedule prepared by an independent statistician. Patients, investigators, study teams and the sponsor Alizé Pharma were unaware of the group assignments. Patients were admitted to the study center for study assessments performed on Day -1 (baseline), on Day 1 (first dose) and Day 14 (last dose). Patients were discharged from hospital following study assessments and were resident at home during the study period (named here home-residents). At Hendaye site,France, a PWS-dedicated unit that provides personalized management strategy including weight control, patients were resident at the hospital during the whole study period.

Data were collected at each site and statistical analyses were performed by ICTA PM (Dijon, France) and Excelsus Statistics (Montréal, Canada).

### Participants

Eligible participants were male and female subjects with genetically confirmed PWS established by standard DNA methylation test or fluorescent in situ hybridization, and evidence of hyperphagia as judged by the investigator. Severity of hyperphagia was scored at baseline (week 0) using a 7-point severity scale. Individuals aged 18 to 40 years inclusive were recruited at first-stage. The upper age limit was subsequently raised to 50 years in order to have a study population as representative as possible of the general adult PWS population. Following review by an independent Data Monitoring Committee of safety data for the first 20 adult patients, inclusion of subjects aged 12 to 17 years was allowed. Growth hormone treatment was permitted if doses had been stable for at least 1 month prior to screening. Key exclusion criteria were history of chronic liver disease, history of acute or chronic pancreatitis, type 1 diabetes, use of insulin, use of GLP-1 analogs, use of weight loss agents or drugs known to affect appetite within 2 months prior screening, and co-morbid condition or disease including respiratory disease or psychiatric disorder diagnosed less than 1 month prior to screening. Written consent was obtained from patients, parents or legal guardians, as appropriate.

### Assessments

Adverse events (AEs) were collected from the signature of the informed consent until the follow-up visit performed on Day 28. In accordance with Good Clinical Practice guidelines and applicable local regulatory requirements, AEs were recorded on a standardized Adverse Event form that was included in the patient’s electronic case report form, and described in detail. Physical exam, measurement of vital signs and laboratory tests including hematology, biochemistry, coagulation, and urinalysis were carried out within 7 days prior to Day 1 (first dosing day) and on Day 14. Laboratory tests were performed at local site laboratories. Follow-up on AEs was performed on Day 28. Body weight (BW) and waist circumference (WC) were obtained from patients on Day-1 and Day 14. WC was measured using a standard anthropometry procedure [31]. Fat mass was added to the study assessments subsequent to the trial commencement but before finalization of the statistical plan to further evaluate changes in body composition. Fat mass data is therefore only available for a subset of patients. Assessment was performed on Day-1 and Day 14 using bio-impedance (TANITA BC-418MA, Japan, or OMRON BF-310, OMRON Healthcare Co. Ltd., Japan).

Food-related behaviors were assessed using the Hyperphagia Questionnaire (HQ), a multi-item disease-specific instrument widely used in clinical practice that has been specifically designed, developed and validated to capture food-related behaviors in PWS as reported by care providers [32]. Psychometric properties of the HQ have been explored in the context of this study and data showed that the instrument was valid and reliable. The questionnaire was filled in on Day-1 (baseline) and Day 14. Items were rated on a 5-point scale (1 = not a problem to 5 = severe and/or frequent problem). The following scores were calculated by summing the following items: a) total score (items 1 to 11), b) behavior domain (items 2, 4, 5, 8, 10), c) drive domain (items 1, 3, 6, 9) and d) severity domain (items 7, 11) scores. At the time of the conception and design of the study, there was no established efficacy endpoint for interventional clinical trials in hyperphagia and food-related behaviors associated with PWS. A 9-item score (items 1, 2, 4–9, 11) has been subsequently added and is presented here Consistent with discussion the study sponsor and other sponsors had with the US Food and Drug Administration, item 3 (effort required to redirect) and item 10 (cleverness in obtaining food) have been removed because they do not comply with FDA guidance on clinical outcome measures that support pharmaceutical drug development (limit concepts to behaviors observable by caregiver) [33, 34]. The HQ is provided as supplementary information (see S1 Study Protocol).

Feelings of appetite were captured using a Patient-Reported Outcome (PRO) instrument designed for capabilities of patients with PWS and that has been shown to be valid and reliable, as documented by psychometric evaluations including qualitative interviews. Patients were asked to indicate how much food they could eat by choosing from among six responses labeled from “nothing at all” to “a very very large quantity”. Responses were associated with cartoons representing increasing amounts of food items. Patients were trained and acclimated to the PRO instrument prior to randomization and were tested on Day-1, Day 1 and Day 14 prior and after a standardized isocaloric meal (mean number of calories: 313, mean composition for carbohydrates: 61%, lipids: 22%, and proteins: 17%). Appetite scales were administered immediately following meal and 2 hours following meal. Patients’ responses were scored from 0 to 5 before analysis.

Blood samples for measurements of glucose, insulin, AG and UAG were collected at 30 min and 180 min post-start of breakfast on Day -1, Day 1 and Day 14 after an overnight fast. Plasma glucose and serum insulin were determined on Cobas^®^ analyzers (Roche Diagnostics) at Biomnis Laboratories (France) by a colorimetric enzymatic method and electrochemiluminescence immunoassay (ECLIA), respectively. AG and UAG levels were determined on esterase inhibitor-treated plasma samples (with AEBSF) using two-step double-antibody sandwich enzyme immunoassays, obtained from SPIBio (Bertin Pharma, France; A05306 and A05319, resp.). The limit of detection for AG and UAG is 4 and 6 pg/mL, respectively. Intra-assay and inter-assay variations are lower than 15% for the two assays.

### Statistical analyses

A number of 20 patients per group was considered appropriate for initial evaluation of safety and efficacy on eating behaviors of AZP-531 in this rare patient population. Statistical analyses were performed on the full analysis set that included all randomized patients who received at least one dose of study drug. Adverse events, safety laboratory tests and vital signs were presented descriptively. For other variables, comparisons of treatment versus placebo at Day 14 were performed using ANCOVA models with baseline Day-1 as covariate. The models included appropriate factors such as treatment and genetic subtype in addition to the covariate. For HQ, analyses excluded one outlier patient (i.e. studentized residual outside the -3 to 3 range) and supportive non-parametric Mann-Withney tests were performed. Effect size and 95% confidence intervals (CI) were also derived from the ANCOVA using the ESTIMATE statement in SAS^®^. Between-days comparisons were performed using repeated analysis models (based on the MIXED SAS^®^ procedure with a REPEATED statement) that included appropriate factors such as day, treatment, and genetic subtype. An appropriate variance-covariance matrix was chosen (based on the corrected Akaike information criterion (AICC)) to model the dependent variable through time within one subject. All tests were two-sided (at α = 0.05). Because of the environment difference between Hendaye and other sites, prespecified analyses were performed on HQ in both all patients and in patients that were home-residents (i.e. excluding Hendaye patients). Repeated ANOVA on the change from baseline for glucose and insulin were performed on the full set (including effects size and 95% CI from the ESTIMATE statement in SAS^®^) and patients identified as having impaired glucose tolerance (IGT) or type 2 diabetes (T2D). Pearson’s coefficient of correlations were calculated to determine the relationship of the changes from baseline to Day 14 in the HQ scores (total and 9-item) and in the glucose. Cumulative distribution function (CDF) of patients with a change in the total HQ calculated score from baseline to Day 14 was examined to further characterize the treatment effect. As distribution of the HQ data suggested a floor effect, the change in the HQ 9-item scores was also presented descriptively in home-residents that displayed a baseline HQ 9-item score≥19 which was considered acceptable to demonstrate a meaningful treatment effect. Statistical analyses were performed using SAS^®^ software version 9.2 or higher.

## Results

### Patients

**Fig 1:**
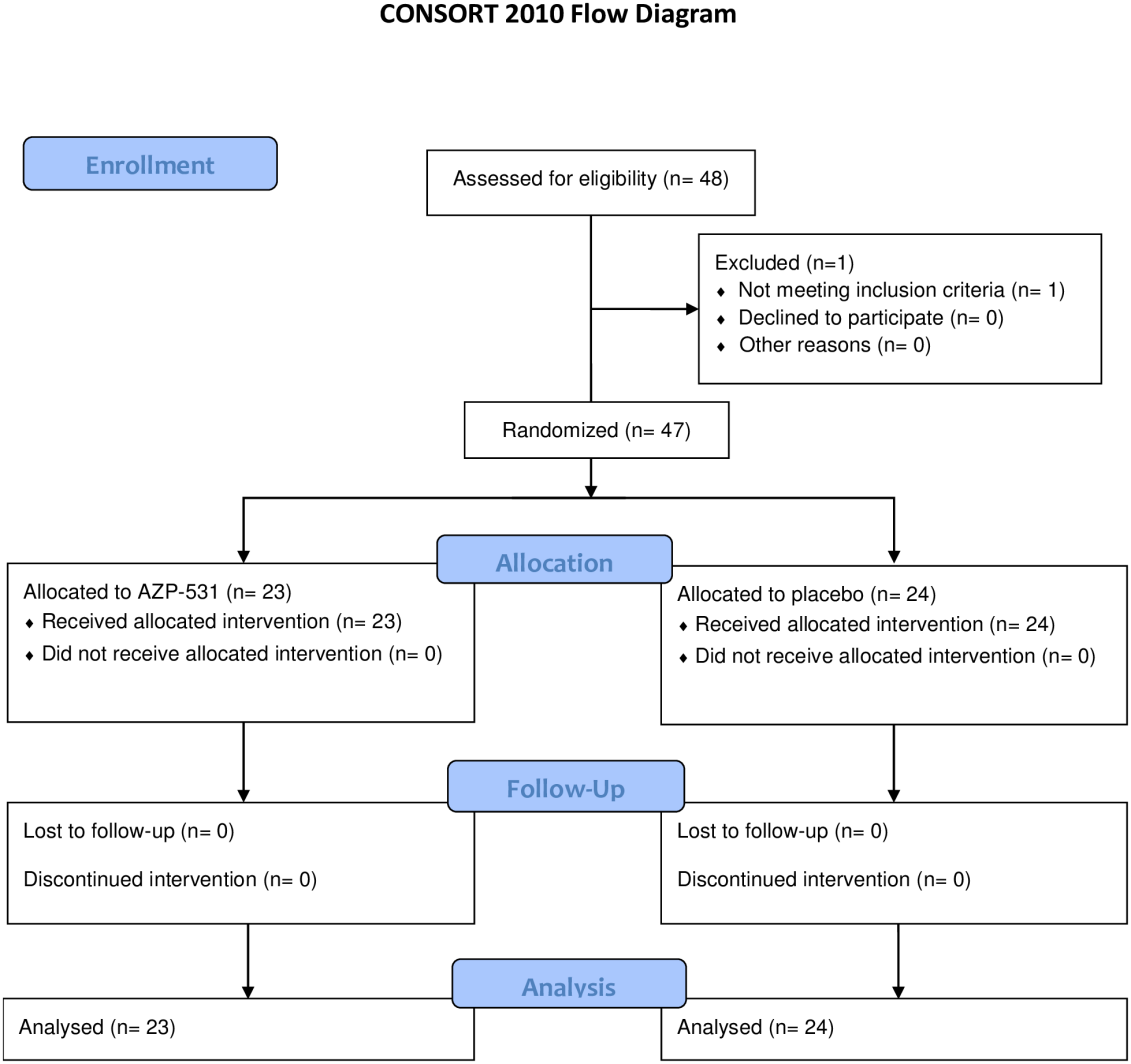
Enrollment and outcomes.

Of 48 patients who were screened, 47 patients underwent randomization (one patient did not meet eligibility criteria) of whom 23 were assigned to receive AZP-531 and 24 to receive placebo (Fig 1). In AZP-531 and placebo groups, there were 19 and 20 home-resident patients, respectively. All randomized patients completed the treatment and follow-up periods. Three adolescents, aged 13, 17 and 17 years, participated in the study and were all in the AZP-531 group. Baseline characteristics were comparable between groups with the exception of the male to female ratio that was higher in the AZP-531 group, although males and females were equally represented in the study (Table 1). Genetic subtype, Intelligence Quotient, and AG/UAG ratio data were consistent with published data [25, 35, 36]. All patients were hyperphagic, as evidenced by the HQ scores and a hyperphagia severity score scale, and were considered in nutritional phase 3 based on Miller’s classification [2]. Adult patients had a BMI ranging from 24.2 to 67.4 kg/m^2^, 23% of them were not obese based on BMI cut-off. BMI was expressed in Standard Deviation Score for the 3 adolescents with values at 1.1, 0, -0.5. Twenty-three percent of the adult study population had either IGT or T2D, also in line with published data [37].

**Table 1:** Baseline characteristics.

Characteristic	AZP-531 N = 23	Placebo N = 24	All N = 47
**Age (y)**	28.0 ± 7.7	25.7 ± 5.6	26.8 ± 6.7
**Median (min-max)**	26.6 (13–46)	24.4 (19–38)	25.5 (13–46)
**Age group**			
<**18 years, n**	3	0	3
≥ **18 years, n**	20	24	44
**Male:Female ratio**, %	65:35	33:67	49:51
**Deletion:non deletion**^a^, %	74:26	67:33	70:30
**IQ**^b^	61.4 ± 12.0	69.8 ± 21.0	65.6 ± 17.3
**Median (min-max)**	61.0 (45–87)	65.0 (47–127)	62.0 (45–127)
**Weight, kg**	87.7 ± 23.6	99.7 ± 26.4	93.8 ±25.5
**Median (min-max)**	87 (53–138)	91 (65–161)	88 (53–161)
**BMI (kg/m^2^)**			
**All patients**	35.1 ± 11.8	40.8 ± 11.8	38.0 ± 12.0
**Median (min-max)**	32.4 (20.6–64.6)	38.4 (27.1–67.4)	34.0 (20.6–67.4)
≥ **18 years**	37.1 ± 11.4	40.8 ± 11.8	39.1 ± 11.6
**Median (min-max)**	33.7 (24.2–64.6)	38.4 (27.1–67.4)	34.2 (24.2–67.4)
**WC (cm)**	106.6 ± 17.2	115.2 ± 18.5	111.0 ± 18.2
**Median (min-max)**	106 (80–139)	111 (92–154)	107 (80–154)
**Hyperphagia severity score (/7)**	4.52 ± 0.99	4.46 ± 1.38	4.49 ± 1.20
**HQ total score (/55)**^c^	30.1 ± 9.2	28.5 ± 7.8	29.3 ± 8.5
**HQ 9-item score (/45)**	23.1 ± 7.7	21.2 ± 6.6	22.1 ± 7.2
**Current use of GH, n**	7	4	11
**Current use of sex hormones, n**	9	10	19
**Fasting glucose (mmol/L)**	5.15 ± 0.54	5.09 ± 0.67	5.12 ± 0.60
**Median (min-max)**	5.08 (4.3–6.3)	4.97 (4.3–7.7)	4.98 (4.3–7.7)
**Fasting insulin**^d^ **(pmol/L)**	77.56 ± 56.18	83.45 ± 42.33	80.58 ± 49.08
**Median (min-max)**	56.32 (24.4–242.5)	68.88 (38.0–181.5)	62.42 (24.4–242.5)
IGT and T2D, n	5	5	10
Fasting AG^e^ (pg/mL)	84.81 ± 37.27	119.22 ± 86.96	102.01 ± 68.37
Fasting UAG^f^ (pg/mL)	106.87 ± 46.42	158.06 ± 100.28	132.46 ± 81.48
Fasting AG:UAG ratio^d^	0.77 ± 0.24	0.76 ± 0.33	0.77 ± 0.29

Min-max, minimum-maximum; BMI, body mass index; HQ, hyperphagia questionnaire; GH, growth hormone; IGT, impaired glucose tolerance; IQ, intelligence quotient (specific test not performed in the context of this study, scores were collected only if available in the subjects’ medical history); T2D, Type 2 diabetes; WC, waist circumference. Data are expressed as mean ± Standard Deviation (SD) or number, unless stated otherwise.

^a^The non-deletion genetic subtype includes uniparental maternal disomy and imprinting defects (when DNA methylation was used for genetic diagnosis additional testing was performed to distinguish deletion from non-deletion subtypes).

^b^Intelligence quotient was obtained from 14 patients in each group.

^c^HQ total score was obtained from 23 patients in each group.

^d^Fasting insulin was obtained from 22 patients in the placebo group.

^e^AG and AG:UAG ratio were obtained from 22 patients in each group.

^f^UAG was obtained from 23 patients in each group.

### Safety

Overall, 28 patients (59.6%) experienced at least one AE during the study (Table 2). The proportion of patients with AEs was comparable between AZP-531 and placebo treatment groups (60.9% versus 58.3%, respectively). The most common AEs in both groups were observed at the injection site with a higher frequency in the placebo group. The majority of AEs was mild in severity and resolved. The number of events was higher with AZP-531 versus placebo, mainly due to repeated injection site pain events observed in one single patient. There were no serious adverse events or AEs leading to treatment discontinuation, and no hypoglycemia events. There were no significant findings with respect to vital sign parameters and safety laboratory tests.

**Table 2:** Adverse events. Data are expressed in number (and percentage) of patients with adverse events.

Event number of patients (%)	AZP-531 N = 23	Placebo N = 24
Any event	14 (60.9)	14 (58.3)
Related event	8 (34.8)	10 (41.7)
Events reported in >5% of the patients		
Injection site erythema	1 (4.3)	2 (8.3)
Injection site hematoma	3 (13.0)	6 (25.0)
Injection site pruritus	0 (0)	2 (8.3)
Oropharyngeal pain	0 (0)	2 (8.3)
Somnolence^a^	2 (8.7)	0 (0)
Events reported in <5% of the patients		
Ear pain	1 (4.3)	0 (0)
Vision blurred	1 (4.3)	0 (0)
Abdominal pain	1 (4.3)	0 (0)
Diarrhoea	1 (4.3)	0 (0)
Asthenia	1 (4.3)	0 (0)
Fatigue	0 (0)	1 (4.2)
Feeling cold	1 (4.3)	0 (0)
Hunger	1 (4.3)	0 (0)
Injection site pain	1 (4.3)	0 (0)
Injection site paresthesia	1 (4.3)	0 (0)
Malaise	1 (4.3)	0 (0)
Erysipelas	1 (4.3)	0 (0)
Gastroenteritis	1 (4.3)	0 (0)
Nasopharyngitis	1 (4.3)	0 (0)
Otitis Media	1 (4.3)	0 (0)
Pharyngitis	0 (0)	1 (4.2)
Rhinitis	0 (0)	1 (4.2)
Fall	0 (0)	1 (4.2)
Ligament sprain	1 (4.3)	0 (0)
Scratch	1 (4.3)	0 (0)
Blood TSH decreased	0 (0)	1 (4.2)
Hyperglycaemia	1 (4.3)	0 (0)
PICA	1 (4.3)	0 (0)
Arthralgia	0 (0)	1 (4.2)
Back pain	0 (0)	1 (4.2)
Pain in extremity	0 (0)	1 (4.2)
Torticollis	1 (4.3)	0 (0)
Dizziness	1 (4.3)	0 (0)
Headache	1 (4.3)	0 (0)
Anxiety	1 (4.3)	1 (4.2)
Nightmare	1 (4.3)	0 (0)
Polyuria	1 (4.3)	0 (0)
Ecchymosis	0 (0)	1 (4.2)
Pruritus	1 (4.3)	0 (0)

^a^Patients who reported somnolence were 23 and 30 years old respectively.

### Food-related behaviors

**Fig 2:**
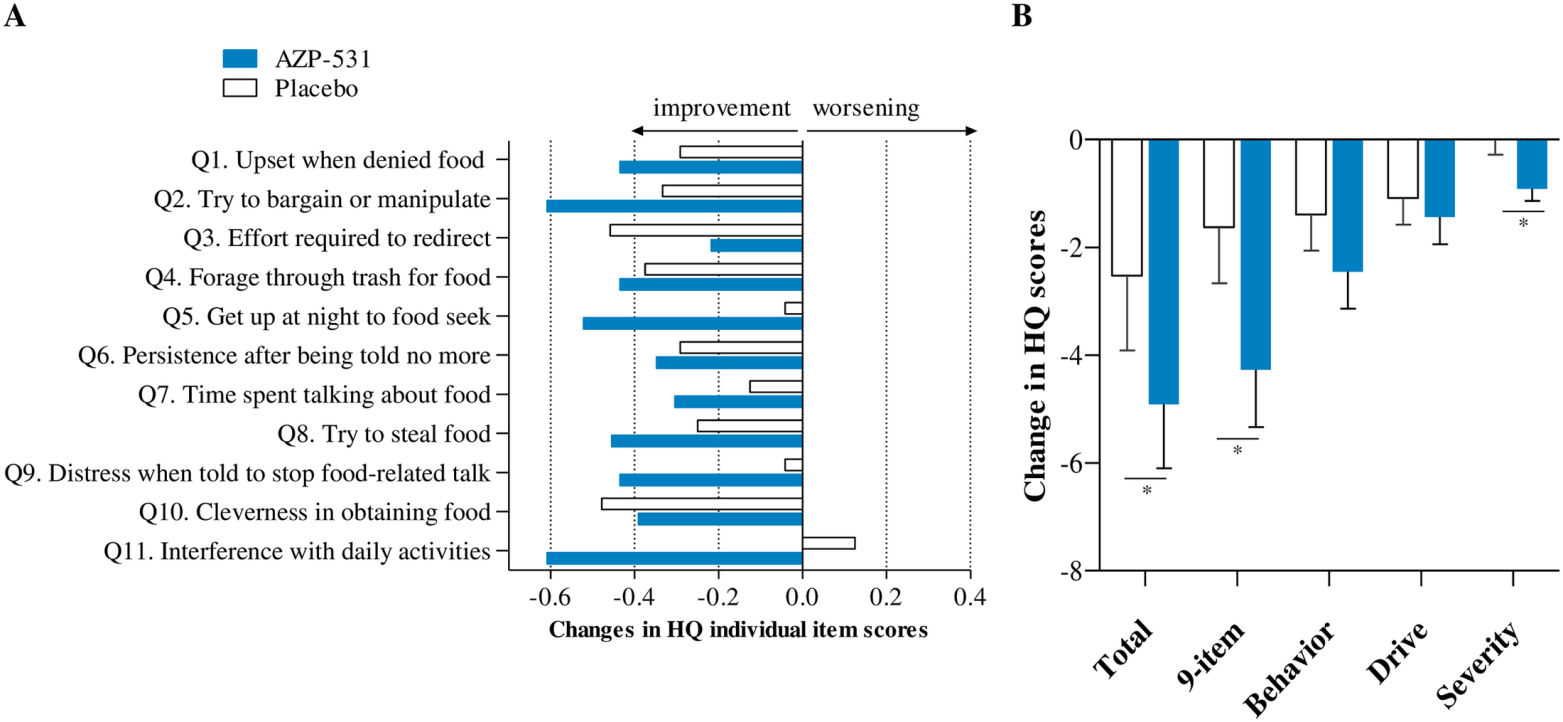
Changes in Hyperphagia Questionnaire individual items and calculated scores from baseline to Day 14. Panels A and B show the mean values of each individual item and each calculated score, respectively. The 9-item score excluded item 3 and item 10 as both items were considered to reflect impression of caregiver rather than capturing concept of interest. A reduction in the score indicates improvement. Bars indicate the standard error. *p < .05 versus placebo.

**Fig 3:**
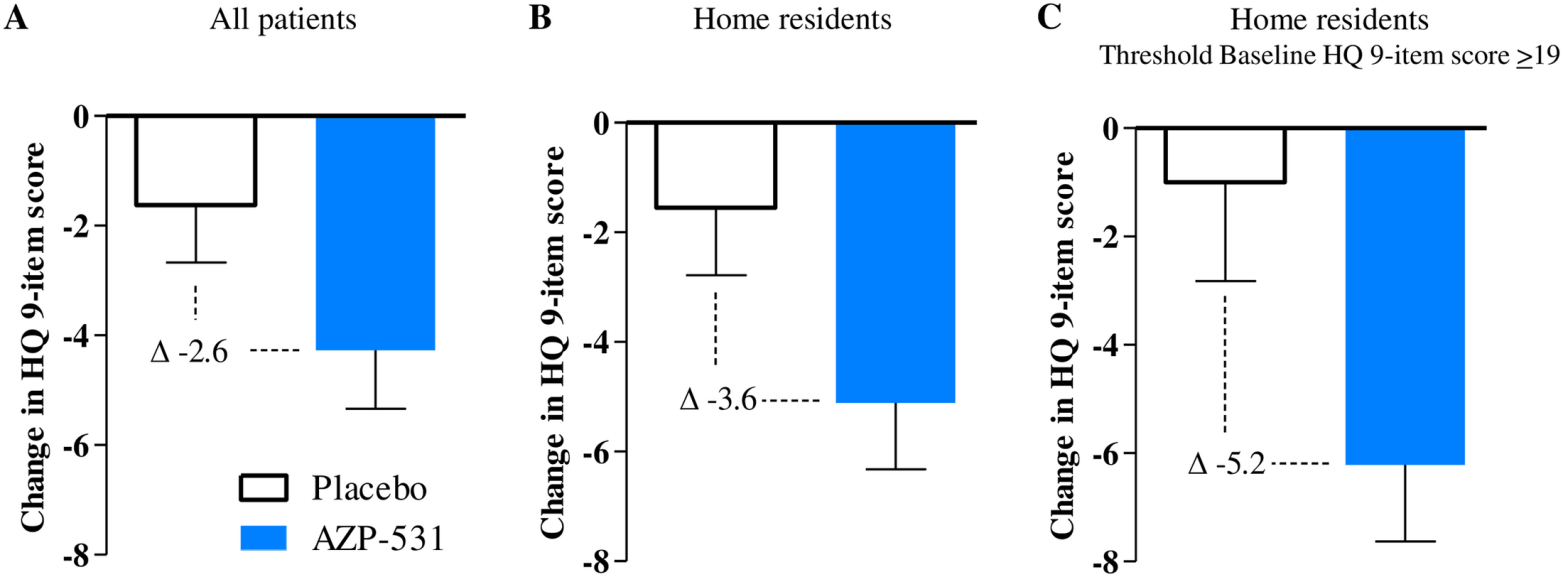
Changes in Hyperphagia Questionnaire 9-item score from baseline to Day 14. Panels A, B, and C show the mean change from baseline in the Hyperphagia Questionnaire 9-item score for all patients (N = 47), home-resident patients (N = 38) and home-resident patients with a threshold base line HQ 9-item score ≥19 (N = 26). A reduction in the score indicates improvement. Bars indicate the standard error.

**Fig 4:**
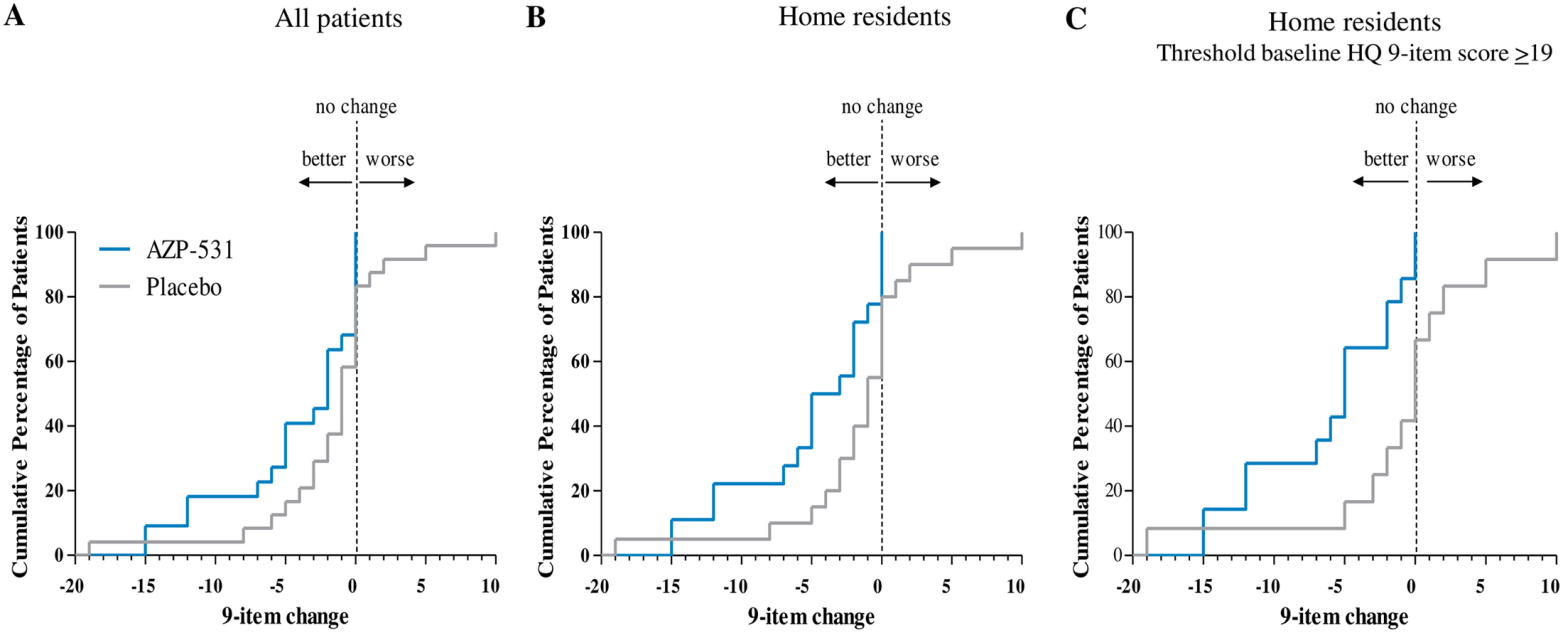
Cumulative distribution function of the Hyperphagia Questionnaire 9-item score from baseline to Day 14. The percentage of treated participants was plotted against the change in Hyperphagia Questionnaire 9-item score from baseline to Day 14.

Globally, mean values of each individual items of the HQ appeared to decrease more with AZP-531 than with placebo (Fig 2A), resulting in decreases in the calculated scores that were more pronounced for AZP-531 as compared to placebo (Fig 2B). The severity domain score decreased in the AZP-531 group only (Fig 2B). This effect was statistically significant versus placebo (p < .05) for the total score (absolute adjusted mean scores of 24.01 versus 27.56, p = .0407, treatment effect 95% CI -6.94; -0.16), the 9-item score (17.42 versus 21.03, p = .0146, 95% CI -6.47; -0.75) and the severity domain score (4.06 versus 4.99, p = .0185, 95% CI -1.69; -0.16). For home-residents, this effect was even larger versus placebo for the total score (absolute adjusted mean scores of 24.02 versus 28.57, p = .0297), the 9-item score (17.43 versus 22.08, p = .0092) and the severity domain score (4.02 versus 5.12, p = .0198). Moreover, supportive non-parametric tests provided similar conclusions. Statistical significance was not reached for the behavior and drive domain scores in all patients (p = .14 and p = .09, respectively) as well as in home-residents (p = .07 and p = .14, respectively). Improvements in the total and 9-item scores were significantly larger in AZP-531 treated patients who had higher scores at baseline (r = -0.5, p = .02 and -0.55, p = .008, respectively). No such significant correlation was found for placebo treated patients (r = -0.04, p = .87 and-0.05, p = .83). As a result, the between-group difference in the 9-item score change was gradually higher in home resident patients, in home resident patients with a baseline score ≥19 as compared to all patients (Fig 3). CDF plots of the change in 9-item HQ score indicate that the percentage of participants showing improvement was consistently higher in the AZP-351 treatment group than in the placebo group. This finding was even more pronounced in home-residents and in those who displayed a HQ score at baseline ≥19 (Fig 4). In addition, no patient in AZP-531 group had a worsening of the 9-item HQ score while a worsening was observed in the placebo group (17%, 20% and 25% of all patients, home-residents and home resident patients who have a HQ score at baseline>19). Comparable CDF plots were observed for the total score.

### Appetite feelings

Overall, mean scores of patient-reported feelings of appetite decreased immediately following meal and returned to baseline 2 hours following meal. At Day 14, values were comparable between AZP-351 and placebo groups for all time points. Day 14 versus baseline comparisons revealed a significant decrease in scores for AZP-531 after breakfast (-1.1, p = .0004) while no significant change versus baseline was noted for placebo (-0.5, p = .10). Values for each treatment group were comparable to baseline at 120 min post-start of breakfast.

### Body composition

Descriptive values for the mean change from baseline to day 14 in study variables are presented in Table 3. BW did not change significantly at Day 14 in both groups while WC decreased by -1.14 cm for AZP-531 (p = .047) versus -0.21 cm for placebo (p = .55). Fat mass also decreased at Day 14 as compared to baseline for both groups, with a change of -1.42% for AZP-531 (p = .046) and of -0.48% for placebo (p = .52). No significant difference between groups was found for body composition.

**Table 3:** Changes from baseline to Day 14 in study variables.

Variable	AZP-531 N = 23		Placebo N = 24	
	n	Mean ± SD	n	Mean ± SD
**HQ scores**				
Total	22	-4.9 ± 5.6^a^	23	-2.5 ± 6.7
9-item	22	-4.3 ± 5.0^a^	24	-1.6 ± 5.1
Behavior domain	22	-2.5 ± 3.2	23	-1.4 ± 3.2
Drive domain	23	-1.4 ± 2.4	24	-1.1 ± 2.4
Severity domain	23	-0.9 ± 1.1^a^	24	0.0 ± 1.6
**Appetite scores at breakfast**				
**Fasting**	23	0.0 ± 1.3	24	-0.3 ± 1.3
**Immediately after**	23	-1.1 ± 1.8^b^	24	-0.5 ± 1.8
**2 hours after**	23	-0.3 ± 1.5	24	-0.2 ± 1.8
**Body composition**				
**Body weight (kg)**	23	-0.07 ± 1.61	24	-0.50 ± 2.25
**Fat mass (%)**	14	-1.42 ± 3.25^c^	14	-0.48 ± 1.48
**WC (cm)**	22	-1.14 ± 2.03^c^	24	-0.21 ± 3.58
**Glycemic measures at breakfast**				
Glucose (mmol/L)				
All patients				
Fasting	22	-0.06 ± 0.20	24	0.04 ± 0.30
At 60 minutes	22	0.17 ± 1.35	24	0.05 ± 1.16
At 180 minutes	21	-0.23 ± 0.74^a^	22	0.15 ± 0.41
IGT/T2D patients				
Fasting	5	-0.03 ± 0.19	5	0.17 ± 0.27
At 60 minutes	5	-0.53 ± 1.78	5	0.74 ± 0.60
At 180 minutes	5	-0.84 ± 1.14	4	0.00 ± 0.35
Insulin (pmol/L)				
All patients				
Fasting	21	2.58 ± 35.30	21	3.73 ± 26.69
At 60 minutes	21	2.73 ± 166.03	22	30.14 ± 240.51
At 180 minutes	19	-3.30 ± 78.57	23	30.28 ± 90.33
IGT/T2D patients				
Fasting	5	23.39 ± 23.75	4	9.33 ± 34.51
At 60 minutes	5	-74.62 ± 142.64	4	80.58 ±79.50
At 180 minutes	3	-63.86 ± 13.70	5	-8.47 ± 47.50
**AG:UAG ratio at breakfast**				
Fasting	21	0.00 ± 0.20	22	-0.03 ± 0.30
At 60 minutes	19	0.04 ± 0.20	21	-0.01 ± 0.19
At 180 minutes	19	0.05 ± 0.49	20	-0.13 ± 0.45

HQ, hyperphagia questionnaire; IGT, impaired glucose tolerance; T2D: Type 2 diabetes. Data are expressed as mean ± SD.

^a^p < .05 as compared to placebo (ANCOVA excluding one outlier patient).

^b^p < .001 as compared to baseline (ANOVA).

^c^p < .05 as compared to baseline (ANOVA).

### Glycemic measures

**Fig 5:**
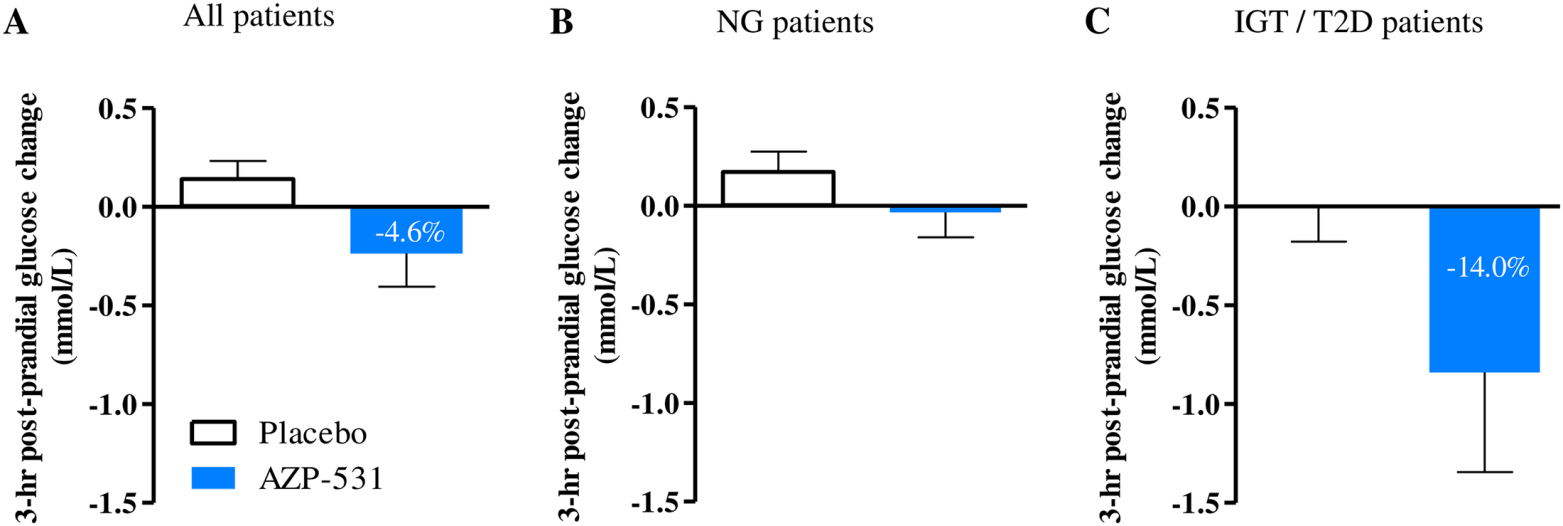
Post-prandial glucose change in treated patients from baseline to Day 14. Mean values of the 3-hour post-prandial glucose change from baseline to Day 14 was plotted for all treated patients (Panel A), normoglycemic (NG) patients (Panel B), and patients with Impaired Glucose Tolerance or Type 2 Diabetes (IGT/T2D) (Panel C).

There was an overall (Day 1 and Day 14 combined) significant reduction in blood glucose levels versus baseline at 180 minutes post-start of breakfast for AZP-531 vs. placebo (change-from-baseline adjusted means of -0.21 versus 0.18 mmol/L, p = .008, treatment effect 95%CI -0.68; -0.11) with p = .05 (95% CI -0.79; 0.00) at Day 1 and p = .04 (95% CI -0.75; -0.02) at Day 14. The reduction appeared greater in AZP-531 patients with IGT or T2D. The decrease in glucose was significantly larger in AZP-531 patients who had higher baseline glucose values (r = -0.47, p = .03) while such a significant correlation was not seen in placebo (r = -0.17, p = .46) (Fig 5). In parallel, there was an overall (Day 1 and Day 14 combined) significant effect for a decrease in insulin levels in AZP-531 versus placebo at 180 min post-start of breakfast (change-from-baseline adjusted means of -22.31 versus 28.48 pmol/L, p = .02, treatment effect 95% CI -91.77; -9.76) with a trend at Day 1 (p = .05, 95% CI -137.11; 0.93) and no significant effect at Day 14 (p = .21, 95% CI -86.89; 19.95).

### Circulating ghrelin

In both groups, mean AG and UAG values decreased following meal as expected. There were no relevant changes following treatment in ghrelin values and the ratio.

## Discussion

In this study, administration of AZP-531 for 2 weeks to patients with PWS was well tolerated which corroborates safety data from previous AZP-531 studies in humans where no negative safety signals were noted following 2 weeks of continuous treatment at doses up to 60 μg/kg/day in obese subjects and patients with type 2 diabetes [30]. With AZP-531 treatment, there was a significant improvement in food-related behaviors as reported by caregivers using the HQ. In the absence of defined clinical endpoints to assess drug efficacy against hyperphagia, there has been tremendous effort made in the past few years by the academia and the pharmaceutical industry to develop and validate clinical outcome measures. The HQ designed by Dykens at al. has emerged as a reliable and valid tool and a 9-item modified version of the HQ specifically for use in clinical trials (HQ-CT) has been recently developed incorporating industry guidance related to clinical outcome assessment and FDA recommendations. Based on the dialogue recently engaged between the industry and regulatory agencies, the 9-item HQ-CT score has been recently considered as a relevant clinical outcome measurement from a drug development perspective. In this study, the 9-item score calculated with the same items as the HQ-CT significantly improved by 2.6 versus placebo after only 2 weeks of treatment. In addition, post-meal appetite scores were shown to be reduced as compared to baseline with AZP-531 and not with placebo, suggesting improved satiety following treatment with AZP-531 and supporting the food-behavior data.

In our study, the improvement for AZP-531 vs placebo in the HQ 9-item score appears larger in home-resident patients as compared to the whole study population because of lesser effect especially in foraging and food stealing behaviors in hospital-residents which is not unexpected in such a supervised and controlled setting. Distribution of the data also indicates that a significant proportion of patients show scores close to the lowest values, suggesting a floor effect. This is further demonstrated when a threshold HQ score was applied to the baseline data (≥19) which resulted in a treatment effect of 5.2 versus placebo in these methodologically optimized conditions. This points to the appropriateness of such a methodological approach for the capture and analyses of HQ data in future development.

Patients with PWS display aggressive food-seeking behaviors that may result in life-threatening complication such as choking [38] or gastric necrosis and rupture [39]. A 40-year mortality survey recently published shows that these complications are the causes of a significant proportion of deaths in this patient population [6]. In addition, patients with PWS always have an increased body fat even when non obese and carry a significant risk of developing cardio-vascular and metabolic complications that represent one of the leading causes of mortality in adult patients and contribute to reduced life expectancy [6, 40]. Management of food-related behaviors and obesity is problematic and represents one of the highest priority medical need for which there is today no other option than continuous supervision, strict control over access to food, dietary restriction and regular exercise [41]. Drugs and strategies tested so far including available anorexigenic agents, endocannabinoid antagonists, recombinant human Growth Hormone therapy, and gastric banding or bypass, have been proven ineffective in reducing hyperphagia or were associated with safety issues [42–44]. There are a few pharmacological targets that are currently undergoing testing in randomized controlled trials including oxytocin and analogs, GLP-1 analogs, a melanocortin 4 receptor agonist and a K+-ATP agonist. The clinical development of beloranib, an inhibitor of methionine aminopeptidase 2, has been recently terminated due to safety issues [45].

Data from the largest cohort ever published on ghrelin in PWS have shown that circulating AG levels are elevated in patients with PWS when hyperphagia and obesity develop whereas UAG levels are normal [25], reflecting a relative UAG deficit. This ghrelin pattern appears unique to PWS suggesting an intrinsic defect in the ghrelin regulation in PWS and is opposed to common obesity where AG levels (in addition to UAG levels) are decreased versus healthy controls. The rationale for investigating AZP-531 in patients with PWS was based on experimental evidence indicating that administration of UAG abolished the orexigenic effect of AG in a rat model [12] and that AZP-531 was able to reproduce this inhibitory effect. Further investigation showed that UAG suppresses AG-induced hypothalamic neuronal activity involved in food intake [12] and acts as a functional antagonist of AG. UAG may act independently of AG by affecting expression of melanocortin receptors and stimulating nesfatin neurons, pathways also known to modulate food intake [12, 13].

No effect on circulating ghrelin values and the ratio was noted following 14 days of treatment with AZP-531 in this study. This is a consistent finding as no change in ghrelin levels was observed in previous human studies where healthy subjects, obese subjects and patients with T2D were treated for up to 14 days with AZP-531 [30]. Plasma AG levels were also unchanged following UAG treatment to healthy subjects [46] in contrast to a previous report where decreased AG levels were observed following infusion of UAG to patients with T2D [47]. This latter finding may be explained by an assay issue.

Here, we also report a significant decrease from baseline in fat mass and waist circumference with AZP-531 but not with placebo, with no change in body weight. Furthermore, there was a significant decrease in post-prandial blood glucose versus placebo that was more pronounced in patients with IGT or T2D, with no change or a decrease in insulin levels. This finding appears of clinical relevance as T2D is reported in about 25% of adults with PWS [37]. The above-mentioned results were obtained following only 2 weeks of treatment in patients with heterogeneous body composition data at baseline. Fat mass was assessed by bioelectrical impedance that has limitations as compared to gold standard dual-energy X-ray absorptiometry. Nevertheless, data are consistent with previous findings showing improvement in body composition, glycemic parameters and insulin sensitivity following treatment with UAG or AZP-531 in obesity and diabetes conditions in animals and humans and suggest that AZP-531 reproduces these effects on adipose tissue and insulin sensitivity in patients with PWS. In a high-fat-diet murine model, treatment for 4 weeks with UAG or AZP-531 was shown to prevent glucose intolerance, insulin resistance, and fat accumulation, all these effects being considered as direct peripheral effects of UAG and AZP-531, and were translated into the clinic [11]. A short-term treatment with UAG improved insulin sensitivity in patients with Type 2 diabetes [47]. A 2-week treatment with AZP-531 improved glucose concentrations, without increasing insulin levels, suggesting an insulin sensitizing effect, and decreased body weight in overweight/obese subjects while patients with Type 2 diabetes had their HbA1c reduced [30].

Based on these findings, significant improvement in body composition and metabolic parameters can be reasonably expected in longer-term and larger clinical trials in PWS. This is also supported by epidemiological reports that link relative UAG deficiencies with higher body weight and insulin resistance, while correcting body weight is accompanied by an increase in UAG [48, 49].

In summary, treatment with AZP-531, an UAG analog, for 2 weeks was well-tolerated in such a frail PWS population and resulted in significant improvement in food-related behaviors supported by a reduction in hunger, as well as encouraging metabolic signals. Our data warrant further investigation to assess long-term safety and efficacy of AZP-531 in patients with PWS and hyperphagia for whom no approved treatment is currently available.

## Supporting information

S1 CONSORT Checklist (DOC)

S1 Study Protocol (PDF)
