# Supplementary material for: AZP-531, an unacylated ghrelin analog, improves food-related behavior in patients with Prader-Willi syndrome: A randomized placebo-controlled trial
Source: PLoS One. 2018 Jan 10;13(1):e0190849. doi: 10.1371/journal.pone.0190849 (PMC5761957; doi:10.1371/journal.pone.0190849)
Supplement: S1 Study Protocol — (PDF) [file pone.0190849.s002.pdf]

Final protocol

CLINICAL STUDY PROTOCOL

Protocol Number: AZP01-CLI-002

EudraCT Number: 2014-001670-34

A Phase IIa, randomized, double-blind, placebo-controlled, multi-center study to evaluate the safety, tolerability, and effects of AZP-531, an Unacylated Ghrelin analog, on food-related behavior in patients with Prader-Willi Syndrome

Product: AZP-531

Alizé Pharma

15G Chemin du Saquin

69130 Ecully, France

Tel: +33 472 18 94 28

Fax: +33 478 33 36 29

Amended Protocol

Version 3.3

Release date: May 6, 2015This document contains confidential information belonging to Alizé Pharma. Except as maybe otherwise agreed to in writing, by accepting or reviewing this document, you agree to hold such information in confidence and not disclose it to others (except where required by applicable law) nor use it for unauthorized purpose. In the event of actual or suspected breach of this obligation, Alizé Pharma should be promptly notified.

STATEMENT OF COMPLIANCE

This trial will be conducted in compliance with the protocol, International Conference on Harmonisation Good Clinical Practice E6 (ICH-GCP), the declaration of Helsinki and the applicable national and local regulatory requirements.

PROTOCOL SIGNATURES

I agree with the content of this protocol and the confidential nature of the documentation made as part of this study. I have read the protocol and understand it and will work according to it and to the principles of Good Clinical Practices, applicable laws and regulations, and the Declaration of Helsinki.

INVESTIGATOR

Printed Name	Signature	Date

COORDINATING PRINCIPAL INVESTIGATOR

Printed Name	Signature	Date
MAITHÉ TAUBER		MAY 6, 2015

I agree with the content of this protocol and the confidential nature of the documentation made as part of this study. I have read the protocol and understand it and will ensure that the clinical study is conducted according to it and according to the principles of Good Clinical Practices, applicable laws and regulations, and the Declaration of Helsinki.

SPONSOR MEDICAL REPRESENTATIVE

SORAYA ALLAS	[signature]	**MAY 6, 2015**
Printed Name	Signature	Date

STUDY CONTACTS

SPONSOR

Alizé Pharma

Dr. Soraya Allas

15G Chemin du Saquin
69130 Ecully, France
Phone: +33 472 18 09 24
Cell: +33 6 29 21 73 16
Fax: +33 478 33 36 29
Email: allas@alz-pharma.com

COORDINATING PRINCIPAL INVESTIGATOR

Pr. Maïthé Tauber

Hôpital des Enfants, Centre d'Endocrinologie, Maladies Osseuses, Génétique et Gynécologie Médicale
CHU de Toulouse, 330 Avenue de Grande Bretagne
31059 Toulouse Cedex 9, France
Phone: +33 534 55 85 51
Email: tauber.mt@chu-toulouse.fr

STUDY MANAGEMENT AND MONITORING

ICTA

Primary Contact:

Sébastien Marie

11, rue du Bocage
21121 Fontaine-Lès-Dijon, France
Phone: +33 380 53 40 49 (direct line) / 19 / 00
Fax: +33 380 57 10 22
Email: pws@icta.fr

SERIOUS ADVERSE EVENTS (SAE) REPORTING

ICTA

Françoise Bullier

11, rue du Bocage
21121 Fontaine-Lès-Dijon, France
Phone: +33 380 53 40 00
Fax: +800 53 40 53 40
Email: pharmacovigilance@icta.fr

TABULATED PROTOCOL SUMMARY

**Sponsor Protocol Number**	**AZP01-CLI-002**
**Protocol Title**	A Phase IIa randomized, double-blind, placebo-controlled, multi-center study to evaluate the safety, tolerability, and effects of AZP-531, an Unacylated Ghrelin analog, on food-related behavior in Prader Willi Syndrome patients.
**Protocol Synopsis Version**	3.3 - Final
**Sponsor**	Alizé Pharma<br><br>15G chemin du Saquin<br><br>69130 Ecully, France
**Principal Investigator**	Professor Maïté Tauber<br><br>Hôpital des enfants, CHU de Toulouse<br><br>330 av de Grande Bretagne<br><br>31059 Toulouse Cedex 9, France
**Number of Centers**	Approximately seven centers in France and Western Europe.
**Clinical Phase**	Phase IIa
**Objectives**	**Primary objective**<br><br>The primary objective will be to evaluate the safety and tolerability of one dose of AZP-531 adjusted for bodyweight administered over 2 weeks as daily subcutaneous injection.<br><br>**Secondary objectives**<br><br>The secondary objectives will be to evaluate effects of AZP-531 after 2 weeks of administration on fullness/satiety and appetite/prospective food consumption, as assessed by Numerical Rating Scales (NRS).<br><br>**Exploratory Objectives**<br><br>• Effects of 2 weeks of administration of AZP-531 will be evaluated on the following:<br>○ Food-related behavior as assessed by the PWS hyperphagia questionnaire (global score, domain scores, and individual scores for selected questions)<br>○ Clinical Global and Hyperphagia improvement and/or severity as assessed by the investigator using the Clinical Global Impression (CGI) scale and Clinical Hyperphagia Impression (CHI and CHI) scales<br>○ Blood levels of Acylated Ghrelin (AG) and Unacylated Ghrelin (UAG); AG/UAG ratio<br>○ Blood levels of glucose, insulin, human Pancreatic Polypeptide (hPP), and IGF-1

	○ Food-related anxiety as assessed by Numerical Rating Scale (NRS)<br>○ Overall behavioral and emotional problems as assessed by the Developmental Behavior Checklist 24 (DBC-P24)<br>• Effects of AZP-531 will be also evaluated on fullness/satiety, appetite/prospective food consumption, and food-related anxiety as assessed by NRS performed daily during the ambulatory treatment period<br>• Evaluation of the three NRS used in the study (content validity and measurement properties)
**Study Design**	This study is a Phase IIa randomized, double-blind, placebo-controlled, multi-center study evaluating one dose of AZP-531 administered once daily for 14 days.<br><br>After screening (Day -2), eligible patients will be admitted to the study center and randomized via an Interactive Web Response System (IWRS). Randomization will be 1:1 among treatment arms; stratification will be performed based on genetic subtypes (deletion, non-deletion). Patients will be evaluated on Day -1 for baseline measurements, on Day 1 following administration of study drug and discharged at the end of the day. They will be admitted to the study center again on Day 13 for evaluation of end of treatment period (Day 14). Follow-up will be performed on Day 28 on the phone. A follow-up visit at the study center may be scheduled within 48 hours, at the investigator's judgment.<br><br>The study schematic is as follows:<br>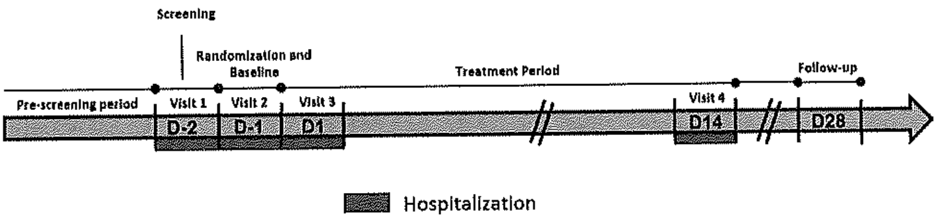<br>Following analysis of unblinded study data and if clinical benefits are observed, AZP-531 will be made available for 14 days for placebo patients. Informed consent should be obtained from legal guardians or from the subjects assisted by their legal representative, as appropriate. Safety data will be collected during this time period.
**Planned Sample Size**	**Interim analysis**<br><br>Up to 20 patients aged 18 to 50 years old (with a minimum of 8 patients treated within each of the AZP-531 and Placebo group) who have completed the treatment period (Day 14) will be part of the interim analysis. An unblinded statistician will follow-up the study completion of the patients to make sure that a minimum of 8 patients treated within each group will be included. Eight (8) treated patients, corresponding to 40% of the patients in the AZP-531 group, are considered sufficient to have an overview of the safety results and identify major outstanding safety issue.<br><br>**Final Analysis**<br><br>Approximately 40 evaluable subjects will be enrolled in the study. A number of 20 patients per group is considered appropriate for initial evaluation of safety and preliminary efficacy of AZP-531 in this patient population.

Main Inclusion Criteria	• Male and female patients with genetically confirmed diagnosis of Prader-Willi Syndrome using standard DNA methylation test or fluorescent in situ hybridization<br>• Patients aged 18 years to 50 years old inclusive<br>• Have evidence of increased appetite or hyperphagia, as judged by the investigator<br>• Patients on well-balanced controlled diet and on a regular exercise program as recommended in common clinical practice for this patient population<br>• Women of Child Bearing Potential (WCBP) must have a negative pregnancy test on admission to the study center<br>• All WCBP, sexually active male subjects, and all partners of subjects should agree to use adequate methods of birth control (diaphragm, condoms with spermicide…) throughout the study and for 30 days after the last dose of study drug<br>• Adequate renal function, defined as serum creatinine ≤1.5 × Upper Limit of Normal (ULN) and urine protein/creatinine ratio <0.2<br>• Adequate hepatic function, defined as total bilirubin ≤1.5 × ULN and Aspartate aminotransferase (AST) and Alanine aminotransferase (ALT) levels ≤3 × ULN<br>• Growth hormone treatment will be permitted if doses have been stable for at least 1 month prior to screening<br>• Psychotropic treatment will be permitted and should be stable at least 1 month prior to screening<br>• Any other treatment including thyroid hormones should be stable for at least 1 month prior to screening<br>• In France, patients must be affiliated to the *sécurité sociale* and must be under guardianship measures<br>• Informed consent signed by the legal guardian or by the subject assisted by his/her legal representative, as appropriate
**Main Exclusion Criteria**	• History of chronic liver disease, such as cirrhosis or chronic hepatitis due to any cause, or suspected alcohol abuse<br>• History of acute or chronic pancreatitis<br>• Type I diabetes<br>• Insulin treatment<br>• Use of weight loss agents or drugs known to affect appetite (including GLP-1 analogs) within 2 months prior to screening<br>• Co-morbid condition or disease (such as respiratory disease or psychiatric disorder) diagnosed less than 1 month prior to screening<br>• Co-morbid condition or disease or abnormal laboratory finding that would in the investigator judgment increase the subject risk to participating in this study and that will not allow the patient to complete the study<br>• History or presence of gastrointestinal, hepatic or renal disease or any other condition known to interfere with the absorption, distribution, metabolism or excretion of drugs<br>• Participation in a clinical trial with an investigational agent within 2 months prior to screening<br>• Clinically significant abnormalities on ECG at screening<br>• Pregnant or lactating woman<br>• History of hypersensitivity to drugs with a similar chemical structure or class as AZP-531 (Acylated Ghrelin and Unacylated Ghrelin)<br>• Unwillingness or inability to follow the procedures outlined in the protocol

**Study Drug, Dose, and Route of Administration**	For practical reasons, a fixed dose (2, 3 or 4 mg) has been selected. Patients will receive the following dose of AZP-531 adjusted for body weight.<br><br>Body weight: 30 to 49 kg → Dose 2 mg; 50 to 70 kg → Dose 3 mg; >70 kg → Dose 4 mg<br><br>For patients <30 kg, dose will be adjusted based on actual body weight (60 µg/kg).<br><br>Active drug and placebo will be provided as lyophilized powder in clear glass vials of 2 mL and kept refrigerated prior to reconstitution and use. Placebo will be mannitol.<br><br>Study drug will be reconstituted daily prior to administration with 0.9% saline and administered 30 minutes prior to breakfast from Day 1 to Day 14 by subcutaneous injection at rotating sites in the abdomen.<br><br>The subject will be injected the study drug at the clinical site on Day 1 and 14, and will have a health professional administer the study drug at home on Day 2 to Day 13. Consequently, study drug will be supplied for use at the study center and home. Medication labels will comply with regulatory requirements.
**Concomitant Medications**	Use of weight loss and anorexigenic agents, including GLP-1 analogs, will not be allowed during the study.<br><br>Growth hormone treatment will be permitted if doses have been stable for at least 1 month prior to screening. Psychotropic treatment will be permitted and should be stable at least 1 month prior to screening. Any other treatment including thyroid hormones should be stable for at least 1 month prior to screening.<br><br>Other treatments may be prescribed by the Investigator if judged necessary for the patient.
**Study Schedule and Assessments**	Before the initiation of the study procedures, contact may be performed on the phone or during a routine medical visit. During this contact, the following may be discussed:<br>• Inclusion/exclusion criteria<br>• Medical history, disease history, demographics and concomitant medications<br>• Study objectives, procedures, and informed consent with the subjects or the legal guardians<br><br>**Visit 1: Screening (Day -2)**<br>• Obtain signed informed consent<br>• Review inclusion/exclusion criteria<br>• Review medical history, disease history, demographics, IQ (if available) and concomitant medications<br>• Perform physical examination, obtain vital signs<br>• Perform urinary pregnancy test<br>• Perform safety lab tests in fasting condition (haematology, clinical chemistry, coagulation and urinalysis)<br>• Perform 12-lead ECG<br>• Administer NRS to patients for training and acclimation purposes<br>• Train parents/guardians for administration of NRS to patients during the ambulatory treatment period<br>• Perform comprehension tests of NRS in study centers located in France (only in France for logistical reasons)

Dose table:

Body weight	30 to 49 kg	50 to 70 kg	>70 kg
Dose	2 mg	3 mg	4 mg

Visit 2: Randomization and Baseline (Day -1)

- Review inclusion/exclusion criteria
- Randomize patients via the IWRS
- Obtain vital signs
- Collect concomitant medication (if applicable)
- Obtain Waist Circumference (WC), Body Weight (BW), fat mass by bio-impedance height, and BMI before breakfast
- Administer NRS at selected time points at breakfast and lunch (before meal, at the end of the meal and 120 min. post-start of meal)
- Record any food item not consumed – No food should be served between breakfast and lunch
- Sample for AG, UAG, insulin, glucose, hPP, before breakfast, and 60 and 180 minutes post-start of breakfast. This later time point sampling must occur before start of lunch. A catheter will be inserted for collection of samples performed on Day-1 and Day 1.
- Sample for AZP-531 antibodies
- The following breakfast time activities must be performed in the following order: NRS, insertion of catheter, sampling for selected measurements, meal.
- Perform the PWS Hyperphagia questionnaire and DBC-P24
- Perform Clinical Hyperphagia Impression Severity scale (CHI-S).
- Record adverse events
- Inform the primary doctor about the inclusion of the patient in the study

Visit 3: Start of Treatment at study centre (Day 1)

- Record concomitant medication (if applicable)
- Administer NRS at selected time points at breakfast and lunch (before meal, at the end of the meal and 120 min. post-start of meal)
- Record any food item not consumed – No food should be served between breakfast and lunch
- Sample for AG, UAG, insulin, glucose, hPP, before breakfast, and 60 and 180 minutes post-start of breakfast. This later time point sampling must occur before start of lunch.
- Sample for IGF-1 in fasting condition (before dosing)
- Administer study drug before breakfast
- The following breakfast time activities must be performed in the following order: NRS, sampling for selected measurements, dosing, meal (meal must be taken 30 minutes following dosing).
- Record adverse events
- Dispense study drug and supplies for treatment on Day 2 to 13
- Dispense patient diary and glucometer kit

Ambulatory Treatment Period (Day 2 to 13)

- Record concomitant medication (if applicable)
- Administer NRS every day before dosing and after completion of breakfast
- Obtain fasting blood glucose (before dosing) using glucometer every day and report value in the patient diary
- Administer daily dose of study drug before breakfast (meal must be taken 30 minutes following dosing)
- Record adverse events (the nurse must stay with the patient until 15 minutes after the injection)
- Complete patient diary

	**Visit 4: End of Treatment Period (Day 14)**<br>• Record concomitant medication (if applicable)<br>• Collect all used and unused study drug vials<br>• Perform study drug accountability and compliance<br>• Review patient diary<br>• Perform physical examination and obtain vital signs<br>• Obtain WC, BW, fat mass by bio-impedance before breakfast<br>• Perform safety lab tests under fasting condition (haematology, clinical chemistry, coagulation and urinalysis)<br>• Perform urinary pregnancy test<br>• Administer NRS at selected time points at breakfast and lunch (before meal, at the end of the meal and 120 min. post-start of meal)<br>• Record any food item not consumed – No food should be served between breakfast and lunch<br>• Sample for AG, UAG, insulin, glucose, hPP, before breakfast and 60 and 180 minutes post-start of breakfast. This later time point sampling must occur before start of lunch. A catheter will be inserted for collection of post-breakfast time points.<br>• Sample for IGF-1 in fasting conditions (before dosing)<br>• Sample for AZP-531 antibodies (before dosing)<br>• Administer study drug before breakfast<br>• The following breakfast time activities must be performed in the following order: NRS, insertion of catheter, sampling for selected measurements, dosing, meal (meal must be taken 30 minutes following dosing).<br>• Record adverse events<br>• Perform the PWS hyperphagia questionnaire and DBC-P24<br>• Perform Clinical Global Impression Improvement (CGI-I), Clinical Hyperphagia Impression Improvement and Severity (CHI-I and CHI-S) scales.<br>• The patient must have the same evaluations as those planned at Day 14 in case of premature treatment discontinuation.<br>**Follow-up Visit (Day 28 ± 3 days)**<br>• Patients, parents or guardians (as applicable) interview to assess adverse events<br>• Patients may be scheduled for a subsequent visit (within 48 hours) for further examination (vital signs, BW and WC as well as physical examination and safety labs tests under fasting condition), as judged by the investigator.<br>• The follow-up must be performed even if the patient has prematurely discontinued treatment, 14 days after the last dose of study drug taken (safety follow-up).<br>**Please refer to Schedule of Assessments (Appendix 1).**
**Data Analysis**	**<u>Interim Analysis</u>**<br>Up to 20 patients aged 18 to 50 years old (with a minimum of 8 patients treated within each of the AZP-531 or placebo) who have completed the treatment period (D14) will be part of the interim analysis. This analysis will be performed on safety data as described below in the Safety section.<br>**<u>Final Analysis</u>**<br>**Safety**<br>Evaluation of safety will be based on patients who have received at least one dose of study medication. Individual and summary statistics including mean, median, standard-deviation (SD) and range will be presented in tabular form by treatment group for vital signs and clinical laboratory data.

	Adverse events will be tabulated and summarised according to the current version of the Medical Dictionary for Regulatory Activities (MedDRA) and presented using the total number of AEs and the total number and percent of patients who experienced an AE per treatment group.<br><br>**Efficacy**<br><br>Summary statistics including mean, median, SD and range will be presented in tabular form by treatment group for efficacy variables and their corresponding change from baseline (Day-1) when appropriate. These variables include NRS, PWS hyperphagia questionnaire, DBC-P24, CGI, CHI and pharmacodynamics parameters (AG, UAG, AG/UAG ratio, glucose, insulin, hPP, IGF-1).<br>Analysis of covariance (ANCOVA) or analysis of variance (ANOVA) will be performed at Day 14 for the afforded mentioned parameters. The model will include the parameter at baseline (when appropriate), the stratification variables and the treatment group. Key interactions will also be included to explore subgroup effect (e.g. Age Group-by-Treatment, Genetic Subtype-by-Treatment).

TABLE OF CONTENTS

STUDY CONTACTS 5

TABULATED PROTOCOL SUMMARY 6

TABLE OF CONTENTS 13

LIST OF ABBREVIATIONS 18

1 Introduction 21

1.1 Background Information on Hyperphagia associated with Prader-Willi Syndrome 21

1.2 Background Information on Ghrelin 22

1.3 NonClinical data with AZP-531 23

1.3.1 Pharmacology 23

1.3.2 Safety Pharmacology and Toxicology 24

1.4 Clinical Findings with AZP-531 25

1.5 Summary and Study Rationale 28

1.6 Risk Assessment and Management 28

2 Study Objectives 30

2.1 Primary Objective 30

2.2 Secondary Objectives 30

2.3 Exploratory Objectives 30

3 Investigational Plan 30

3.1 Overall Study Design 30

3.2 Discussion of Study Design 31

3.3 Rationale for Dose Selection 32

3.4 Rationale for the selection of Efficacy Endpoints for Evaluation of Eating Behaviors 32

3.5 Study Population 33

3.5.1 Inclusion Criteria 33

3.5.2 Exclusion Criteria 33

3.6 Study Treatment 34

3.6.1 Study Drug and Clinical Supplies 34

3.6.2 Reconstitution and Administration Procedures 34

3.6.3 Randomization Procedures 35
3.6.4 Blinding and Code Breaks 35
3.6.5 Drug Accountability 35
3.6.6 Concomitant Medications and Non authorized Medications 36
3.7 Visit Schedule and Assessments 36
3.7.1 Visit Schedule 36
3.7.2 Safety Assessments 39
3.7.2.1 Adverse Events (AE) 39
3.7.2.2 Serious Adverse Events (SAEs) 39
3.7.2.3 Safety Laboratory Evaluations 40
3.7.2.4 Vital Signs 40
3.7.2.5 Physical Examination 40
3.7.2.6 Body Weight 40
3.7.3 Efficacy Assessments 40
3.7.4 Exploratory Efficacy Assessments 41
3.7.4.1 PWS hyperphagia questionnaire 41
3.7.4.2 Clinical Global Impression (CGI) scale and Clinical Hyperphagia Impression (CHI) scales 41
3.7.4.3 Hormonal and other laboratory assessments 41
3.7.4.4 Food-related anxiety 41
3.7.4.5 Developmental Behavior Checklist 41
3.7.4.6 Additional assessments of fullness/satiety, appetite/prospective food consumption and food-related anxiety 41
3.7.4.7 Comprehension tests of NRS 42
3.8 Criteria for Discontinuation 42
4 Statistical Analyses 42
4.1 Determination of Sample Size 42
4.1.1 Interim Analysis 42
4.1.2 Final Analysis 43
4.2 Population Sets 43
4.2.1 Full Analysis Set 43
4.2.2 Safety Set 43
4.2.3 Per Protocol Set 43

4.3 Missing Observations 44
4.4 Description of Statistical Methods 44
4.4.1 Demographic and Baseline Characteristics 44
4.4.2 Evaluation of Safety 44
4.4.2.1 Adverse events 44
4.4.2.2 Safety Laboratory Evaluations 45
4.4.2.3 Vital Signs, Waist Circumference (WC), Weight, Height and BMI 45
4.4.2.4 Physical Exam 45
4.4.3 Evaluation of Efficacy 45
4.4.4 Exploratory Evaluations 46
4.4.4.1 Food-related behavior using the PWS hyperphagia questionnaire (global score, domain scores, and individual scores for selected questions) 46
4.4.4.2 Clinical Global Impression (CGI) scale and Clinical Hyperphagia Impression (CHI) scales 46
4.4.4.3 Blood levels of AG, UAG, hPP, glucose and insulin 47
4.4.4.4 Blood levels of IGF-1 47
4.4.4.5 Food-related anxiety 47
4.4.4.6 Developmental Behavior Checklist (DBC-P24) 47
4.5 Evaluation of the NRS 48
4.5.1 Exploration of NRS content validity: qualitative methods 48
4.5.2 Preliminary assessment of the measurement properties of the NRS 48
4.6 Interim Data Analysis 48
5 Safety-Related Procedures 48
5.1 Definitions 48
5.1.1 Adverse Event 48
5.1.2 Serious Adverse Event and Suspected Unexpected Serious Adverse Reactions . 49
5.2 Reporting of Adverse Events 50
5.3 Reporting of Serious Adverse Events and Suspected Unexpected Serious Adverse Reactions50
5.3.1 Initial SAE reporting 50
5.3.2 SAE follow-up 51
5.3.3 SAE occurring after the study 51
5.4 Categorization of Adverse Events 51

5.5 Causal Relationship Assessment 52
5.6 Reporting of pregnancy 52
6 Ethics and Good Clinical Practice 53
6.1 Institutional Review Board / Ethics Committee 53
6.2 Informed consent 53
7 Data Monitoring Committee 54
8 Administrative Procedures 54
8.1 Changes to the Protocol 54
8.2 Quality 55
8.3 Monitoring Procedures 55
8.4 Recording of Data and Retention of Documents 56
8.4.1 Recording of Data 56
8.4.2 Retention of Documents 56
8.5 Auditing Procedures 56
8.6 Handling of Study Medication 57
8.7 Insurance 57
8.8 Publication of Results 57
8.9 Disclosure and Confidentiality 57
8.10 Discontinuation of Study 58
9 Appendices 59
Appendix 1 60
Schedule of Assessments 60
Appendix 2 63
Declaration of Helsinki 63
Appendix 3 68
NRS for Appetite/Prospective food consumption 68
Appendix 4 70
NRS for fullness/satiety 70
Appendix 5 72
NRS for anxiety 72
Appendix 6 74
PWS Hyperphagia Questionnaire 74

Appendix 7 78
DBC-P24 78
Appendix 8 80
CGI-I, CHI-I & CHI-S 80
Appendix 9 84
Comprehension test interview guideline 84
Reference List **96**

LIST OF ABBREVIATIONS

°C	Celsius Degree
µg	microgram
ADR	Adverse Drug Reaction
AE	Adverse Event
AG	Acylated Ghrelin
AgRP	Agouti-Related Protein
ALT	Alanine aminotransferase
AST	Aspartate aminotransferase
BAT	Brown Adipose Tissue
BMI	Body Mass Index
BW	Body Weight
C_{max}	maximum Concentration
CGI	Clinical Global Impression scale
CGI-I	Clinical Global Impression scale-Improvement
CHI	Clinical Hyperphagia Impression scale
CHI-I	Clinical Hyperphagia Impression scale-Improvement
CHI-S	Clinical Hyperphagia Impression scale-Severity
CPK	Creatine Phosphokinase
CRF	Case Report Form
CRO	Clinical Research Organization
DAG	Des-Acyl Ghrelin
DBC	Developmental Behavior Checklist
DBC-P24	Developmental Behavior Checklist for Pediatric patients (short form)
dL	Deciliter
DMC	Data Monitoring Committee
DNA	Deoxyribonucleic Acid
EC	Ethics Committee
ECG	Electrocardiogram
eCRF	electronic Case Report Form
EPC	Endothelial Progenitor Cells
FA	Full Analysis
FFA	Free Fatty Acid
g	gram
GCP	Good Clinical Practice
GGT	Gamma Glutamyl Transferase

GH	Growth Hormone
GHSR	Growth Hormone Secretagogue Receptor
GLP-1	Glucagon-Like-Peptide 1
GOAT	Enzyme ghrelin O-Acyl Transferase
GPCR	G Protein-Coupled Receptor
HDL	High Density Lipoprotein
hERG	human Ether-à-go-go Related Gene
HFD	High Fat Diet
hPP	human Pancreatic Polypeptide
ICH	International Conference of Harmonisation
IGF-1	Insulin-like Growth Factor 1
IQ	Intelligence Quotient
IRB	Institutional Review Board
ITT	Intent To Treat
IWRS	Interactive Web Response System
kg	kilogram
L	Liter
LDH	Lactate Dehydrogenase
LDL	Low Density Lipoprotein
MedDRA	Medical Dictionary for Regulatory Activities
mg	milligram
mL	milliliter
NOAEL	No Observed Adverse Effects Level
NPY	Neuropeptide Y
NRS	Numerical Rating Scales
OC	Observed Case
PI	Principal Investigator
PP	Per Protocol
PT	Preferred Term
PWS	Prader-Willi Syndrome
RBC	Red Blood Cells
SAE	Serious Adverse Event
SOC	System organ Class
SOP	Standard Operating Procedure
STZ	Streptozotocin
SUSAR	Suspected Unexpected Serious Adverse Reaction

T2DM	Type 2 Diabetes Mellitus
tbc	to be confirmed
TEAE	Treatment Emergent Adverse Event
t_{max}	Time of C_{max}
UAG	UnAcylated Ghrelin
ULN	Upper Limit of Normal
VAS	Visual Analog Scales
WAT	White Adipose Tissue
WBC	White Blood Cells
WC	Waist Circumference
WCBP	Women of Child Bearing Potential

1 Introduction

1.1 Background Information on Hyperphagia associated with Prader-Willi Syndrome

Prader-Willi syndrome (PWS) is a rare disease (In Europe, the prevalence ranges between 1 and 9 per 100,000 – source Orphanet, last consultation June 9, 2014). It is a neurodevelopmental and hypothalamic disorder equally affecting both sexes due to genetic abnormalities that result in the absence of expression of genes at the locus q11-q13 on chromosome 15 (1). Approximately 60-65% of affected individuals have a deletion of the paternal chromosome 15q11-q13 while most of the remaining subjects display a maternal uniparental disomy for chromosome 15. Finally, a small percentage of patients may have abnormalities of the imprinting center or translocation involving chromosome 15.

PWS is associated with dysmorphic features, short stature, Growth Hormone (GH) deficiency, hypogonadism, low calorie expenditure and abnormal body composition with reduced fat free mass and increased fat mass. Cognitive impairments, behavioral disturbances as well as psychiatric disorders are also part of the syndrome and may have, as somatic features, differences as a function of genotype (2). The majority of adult patients are under guardianship measures. In France, adult patients live generally in their family home and the guardianship is often one parent or a family member.

The growing understanding of the natural history of PWS has recently led to the identification of several successive nutritional and eating behavior phases (3) proceeding from poor feeding, through normal eating without and with obesity, to hyperphagia and life-threatening obesity. A significant proportion of adults with PWS is diabetic and obesity-related complications are the first cause of death in this patient population.

In PWS, excessive eating behavior may have some variation in the nature and severity but is clearly a significant, salient and constant feature of the syndrome, observed in all genetic subtypes. Patients with PWS typically display aggressive and obsessive food seeking, food storage, foraging and hoarding that are viewed by some physicians as part of a broader abnormal behavior. All of these represent a lifelong source of distress for patients and families and severely and negatively affect social adaptation, occupational performance and quality of life. Patients live a dependent life, requiring strict control over access to food and continuous care and supervision.

The basis for the abnormal eating behavior observed in PWS is not yet understood, but evidences suggest involvement of appetite hormone disturbances and central neural mechanisms regulating food intake. Reported biological abnormalities include a blunted post-prandial increase of anorexigenic human Pancreatic Polypeptide (hPP) as compared to obese subjects (4) and elevated fasting and post-prandial levels of total ghrelin and orexigenic Acylated Ghrelin (AG) as compared to normal and obese individuals.

Hyperghrelinemia has been reported at all ages, including in the first years of life, preceding the onset of hyperphagia and obesity (5). In adults with PWS, plasma total ghrelin levels were shown to positively correlate with ratings of hunger (6). Following a satiating amount of meal, levels slightly decreased but remained elevated as compared to obese and lean controls (7). These observations suggest that the hormone may be responsible, at least in part, for the excessive eating and hyperphagia observed in this patient population.

Published data on Unacylated Ghrelin (UAG) in PWS is limited. Results of a recent large observational study including 138 patients with PWS indicate that both AG and AG/UAG ratio are elevated in PWS as compared to healthy subjects with a higher AG/UAG ratio in children and young adults with weight gain and/or hyperphagia (Kuppens *et al.*, manuscript in preparation).

There is no current effective pharmacological treatment for hyperphagia and associated abnormal eating behaviors, and the only way to efficiently manage hyperphagia, obesity and related complications is strict control over access to food, low-calorie, balanced diet and regular exercise (1). Growth hormone which has been used since the early 2000's for improvement in growth velocity and body composition has no effect on appetite and overeating (8;9).

1.2 Background Information on Ghrelin

Ghrelin is a 28 amino acid hormone that is produced mainly by the stomach. The acylated form of the hormone, namely Acylated Ghrelin (AG), bears an acyl moiety on the serine 3 amino acid (10). The acylation of ghrelin, performed by the enzyme Ghrelin O-Acyl Transferase (GOAT), confers to the hormone the ability to bind and activate the GH Secretagogue Receptor (GHSR), a G Protein-Coupled Receptor (GPCR). GHSR is present in different areas of the brain including the hypothalamus and the anterior pituitary gland, as well as in peripheral organs and tissues.

AG is a pleiotropic hormone displaying central and peripheral biological activities that contribute to the regulation of key endocrine and non-endocrine functions. The hormone has been initially identified as a growth hormone secretagogue and has next emerged as one of the most powerful orexigenic agent and the only one acting from the periphery in animals and humans (11-13). The latter effect is mainly achieved through GHSR of hypothalamic neurons co-expressing Neuropeptide Y (NPY) and Agouti-Related Protein (AgRP) (14). AG has been also shown to regulate energy expenditure and administration of AG promotes weight gain and adiposity through a stimulation of food intake while decreasing energy expenditure and body fat utilization. In the periphery, AG modulates the pancreatic endocrine function and glucose metabolism, and administration of AG to humans induces hyperglycemia (15;16). AG has also effects on the gut (including gastroprotection, stimulatory effects on gastric acid secretion and gastrointestinal motility), the cardiovascular system (including vasoactive effects and cardioprotective effects) and hormonal systems (including prolactin secretion, thyroid axis, and gonadal axis). It is believed that some peripheral effects of AG could be mediated via a different GPCR than GHSR, yet to be identified.

UAG, also known as Des-Acyl Ghrelin (DAG), is devoid of the acyl moiety and is unable to bind and activate GHSR at physiological concentrations (17). For this reason, UAG was first considered as a degradation product, but the molecule has become more and more recognized as a separate hormone. Effects of UAG are most often reported as counteracting or opposing the effects of AG. The first example is given by the earliest clinical report evaluating the acute effects of administration of both forms of ghrelin. Authors have shown that co-administration of AG and UAG to healthy male subjects counteracted the increase in glucose and decrease in insulin induced by AG while administration of UAG alone had no effect (15). These findings motivated further investigation and a serie of clinical pharmacology studies were performed showing that administration of UAG to healthy subjects and type 2 diabetes patients reduced glucose levels, possibly by improving insulin sensitivity (18;19). In some studies, findings were associated with a reduction in plasma AG levels.

Animal and *in vitro* work have provided further evidence of UAG biological and pharmacological properties. As examples, UAG was shown to prevent diabetes, glucose intolerance, fat mass gain, and lipid accumulation in liver and brown adipose tissue (20). UAG counteracting effects on AG activities were also described both *in vivo* and *in vitro* as preventing AG-inhibition of secretion of anorexigenic hPP, AG-inhibition of glucose uptake in muscle cells, AG-induced glucose output in hepatocytes, or AG-induced food intake (21-25). In addition, protective and/or proliferative properties have been described with β-cells, progenitor and mature endothelial cells, as well as cardiac and skeletal muscle cells; some of these activities are also observed and shared with AG (23;26-30). Work on UAG cell receptor is ongoing and data accumulated to date suggest that UAG acts through a GPCR, distinct from GHSR, which may be shared with AG.

Plasma total ghrelin levels, as well as AG and UAG levels, follow a biologicalrhythm. In normal individuals, there is a preprandial rise, which peaks at meal initiation, followed by a post-prandial decrease to baseline levels within the first hour following meal (31-33). Both forms of ghrelin circulate in plasma but AG is rapidly deacylated to UAG by esterases in blood samples. Early publications on ghrelin essentially report measurement of total ghrelin levels as most available immunoassays were nonspecific. Recent progress made with respect to more accurate and specific measurement of the levels of the 2 hormones and stabilization of plasma by esterase inhibitor immediately following sampling has allowed a better evaluation of the plasma AG/UAG ratio in different subject populations (34;35). It appears that in healthy individuals, plasma UAG levels are higher than AG levels. In obese and insulin-resistant subjects, UAG levels are lower as compared to healthy subjects, resulting globally in an elevated AG/UAG ratio (36). Similarly, an elevated AG/UAG ratio is observed in PWS as AG levels are much higher than those of healthy subjects.

In the past ten years, the ghrelin system has emerged as a pharmacological target for the treatment of a broad variety of diseases. In particular, various pharmacological tools including GHSR antagonists, AG-blocking agents and GOAT inhibitors, have been designed with the objective of antagonizing or blocking the effects of AG on food intake, fat mass, and/or glucose. None of the designed drug candidates has moved to clinical testing, to our knowledge, because of lack of efficacy and unwanted effects observed in animal models (37). Effects observed in both animals and humans suggesting that UAG may act by decreasing AG plasma levels and/or activity have provided the rationale for the design of UAG analogs for the treatment of metabolic diseases including type 2 diabetes and the Prader-Willi syndrome.

1.3 NonClinical data with AZP-531

1.3.1 Pharmacology

For pharmaceutical development purposes, a series of UAG analogs, excluding or including serine 3, the site of acylation, were designed and tested using an *in vitro* β-cell survival assay. Analog UAG 6-13 (AZP-502), an 8 amino-acid peptide that does not contain serine 3, was found to be the smallest and most effective fragment able to mimic effect of full length UAG *in vitro* and *in vivo* (38). In addition, this analog shared same high affinity binding sites as UAG on β-cells and EPCs.

Cyclisation of UAG 6-13 fragment resulted in AZP-531 which has improved stability in human plasma as compared to the linear fragment and increased half-life noted in 2 animal species (rat and dog) (39). Details of pharmacokinetic data are presented in the Investigator's brochure. *In vitro* and *in vivo*, AZP-531 overall exhibits a comparable pharmacological profile as full length UAG (20;38). In particular, AZP-531 has been shown to prevent glucose intolerance, insulin resistance, and fat accumulation. AZP-531 was also shown to counteract the orexigenic effect induced by AG in a rat model. Non-pharmacology data of UAG and analogs are summarized in the table below and detailed in the Investigator's brochure.

Non clinical pharmacological results	Tested Molecule(s)		
▪ Improved pancreatic β-cell survival and proliferation	AZP-531	AZP-502	UAG
▪ Trophic effects on β-cells and restored glucose and insulin levels in STZ-rats		AZP-502	UAG
▪ Improved oxygen consumption rate in myotubes	AZP-531	AZP-502	UAG
▪ Improved glucose and FFA uptake in myotubes and adipocytes		AZP-502	UAG
▪ Reversed glucagon- and AG-related increase of glucose output in hepatocytes			UAG
▪ Reversed AG-suppression of the anorexigenic Pancreatic Polypeptide release in mouse and rat pancreatic islets			UAG
▪ In HFD mice, prevention of insulin resistance, glucose intolerance, body weight gain, fat mass gain, inflammation in WAT and lipid accumulation in BAT	AZP-531		UAG
▪ Reversed AG-induced food intake in rat	AZP-531		UAG
▪ Protection of Endothelial Progenitor Cells from oxidative stress and senescence	AZP-531	AZP-502	UAG
▪ Protection of C2C12 myotubes from oxidative stress	AZP-531	AZP-502	UAG
▪ Improved muscle protection and regeneration following hind limb ischemia in mice			UAG
▪ Reduced reperfusion injury following cardiac Ischemia/Reperfusion in mice	AZP-531		UAG
▪ No "off-target" activity on all the 44 receptors/channels/enzymes tested	AZP-531	AZP-502	

1.3.2 Safety Pharmacology and Toxicology

AZP-531 was inactive on all the 44 receptors/enzymes/channels tested for potential "off-target" activity.

Good Laboratory Practice compliant pre-clinical safety studies including genetic toxicology, safety pharmacology, and 2-week-toxicology studies in rats (aged 10 weeks at start of treatment) and dogs (aged 6 months at start of treatment) have been performed.

No genotoxicity was observed with AZP-531 in the Ames test and the Mouse Lymphoma Assay and also in the *in vivo* micronucleus test which was integrated into the 2-week-toxicology rat study.

No blockade of the human Ether-à-go-go Related Gene (hERG) channel was observed at a dose of 30 µmol/mL. No treatment-related effect on blood pressure, heart rate, and electrocardiogram (ECG) was noted in a telemetry study in the dog. In this study, the subcutaneous injection of AZP-531 was associated with defensive reactions during administration of the dose levels of 1.85 and 5.5 mg/kg. In the rat, AZP-531 had no significant effect on the respiratory function, as assessed by plethysmography, and on the central nervous system, as assessed by the Irwin test and open field observations.

Results from repeat dose toxicity studies indicate that AZP-531 has no significant toxic effects when administered twice a day, 8 hours apart, by subcutaneous injection at daily doses up to 2 x 18 mg/kg (rats) and 2 x 5.5 mg/kg (dogs), these doses were therefore considered as the no observed adverse effect level (NOAEL).

In both species, toxicokinetic data indicate that the maximum concentration (C_{max}) is generally observed at 0.5 to 1 hour following each of the daily administration. No accumulation of AZP-531 was observed between first and last day of dosing (after 14 days of treatment). The increase in systemic exposure was linear and dose-proportional.

In the rat, no treatment-related effects on preliminary assessment of fertility including estrus cycle and sperm analysis were noted. In the dog, granulomatous inflammation associated with accumulation of foreign material, likely to be the test item, was observed at the injection site of a few animals (5 animals out of 18) from the AZP-531 treated groups.

1.4 Clinical Findings with AZP-531

A summary of completed, ongoing or planned studies of AZP-531 are presented hereafter.

A Phase I first-in-man, placebo-controlled, single and multiple ascending dose study (AZP01-CLI-001) has been undertaken in order to evaluate the safety, tolerability, pharmacokinetics and pharmacodynamics of AZP-531 in healthy volunteers (Part A), overweight or obese volunteers (Part B) and patients with type 2 diabetes (Part C).

Parts A and B of this study have been completed and final results are available. In these 2 parts, a total of 33 healthy volunteers has received a single subcutaneous administration of AZP-531 while a total of 24 overweight or obese subjects has been administered AZP-531 for 14 days. Data show that administration of AZP-531 was well tolerated at a dose up to 120 µg/kg (single administration) and 60 µg/kg (multiple administrations for 14 days) with no significant abnormalities with respect to safety laboratory parameters (including hematology, coagulation, blood chemistry and urinalysis), vital signs, EGC (both Parts) and telemetry (Part A only).

In healthy volunteers receiving a single administration of AZP-531, median t_{max} was generally reached at approximately 1 hour after dosing. Mean exposures were proportional to the dose and mean half-lives ranged between 2 and 3 hours. A trend for dose-dependent decrease in the insulin post-prandial peak in the active AZP-531 treatment compared to placebo was observed over doses administered, ranging from 0.3 µg/kg to 120 µg/kg. No significant changes in blood glucose were noted.

Comparable pharmacokinetic parameters were observed on Day 1 and Day 14 in overweight/obese subjects receiving AZP-531 for 14 days. Accumulations in maximum and total exposures were negligible following repeated dosing for 14 days. At doses of 15 µg/kg and above, reductions on glucose levels were observed, more particularly in subjects with elevated post-prandial glucose levels at baseline. The effects increased over time of treatment and were associated with unchanged insulin levels, in line with an insulin-sensitizing mechanism of action. Reductions in body weight averaging -2.6 kg were observed in AZP-531 treated subjects (vs. -0.8 kg for the placebo group)

Part A (healthy volonteers) and B (overweight/obese subjects) final data are presented in details in the Investigator's Brochure.

Overview of clinical studies with AZP-531

Type of Study	Study Identifier	Objective(s) of the study	Study Design and Type of Control	Test Product(s); Dosage Regimen; Route of Administration	Number of Subjects (AZP-531)	Study Population	Duration of Treatment	Study Status
Phase I	AZP01-CLI-001 Part A	Safety PK PD	Randomized Placebo-controlled	2.2 mg vials; 0.3, 3, 15, 30, 60, 120 µg/kg s.c.	44 (33)	Male Healthy Subjects Age: 31 ± 9 years BMI: 24.02 ± 2.10 kg/m²	Single dose	Completed Final results are available
Phase I	AZP01-CLI-001 Part B	Safety PK PD	Randomized Placebo-controlled	2.2 mg vials; 3, 15, 30, 60 µg/kg s.c.	32 (24)	Healthy Subjects overweight/obese Age: 32 ± 12 years BMI: 30.70 ± 2.06 kg/m²	14day-MAD	Completed Final results are available
Phase I	*AZP01-CLI-001 Part C*	*Safety PK PD*	*Randomized Placebo-controlled*	*2.2 mg vials Doses tbc s.c.*	*36 (27)*	*T2DM*	*14day-MAD*	*Ongoing*
Phase IIa	*AZP01-CLI-002*	*Safety PD/Efficacy*	*Randomized Placebo-controlled*	*2.2 mg vials 2, 3 or 4 mg based on bodyweight s.c.*	*40 (20)*	*Prader-Willi Syndrome*	*14 days*	*Ongoing*

1.5 Summary and Study Rationale

The rationale for testing AZP-531, an analog of human UAG, in patients with PWS is the following:

- Acylated Ghrelin (AG) is one of the most powerful orexigenic agent and is the only known factor acting from the periphery to induce appetite (in animals and humans)
- In PWS, appetite hormone abnormalities include low levels of anorexigenic hPP and elevated levels of orexigenic ghrelin (AG) with a high AG/UAG ratio, as compared to normal subjects,
- Total ghrelin levels are positively correlated with ratings of hunger and remained elevated after meal as compared to obese and lean controls,
- *In vitro*, AG inhibits hPP release from pancreas, while UAG counteracts this effect,
- In a rodent model, UAG and AZP-531 have been shown to inhibit food consumption induced by administration of AG,
- Infusion of UAG to type 2 diabetes patients have been shown to decrease plasma AG levels.

Based on the animal and human data obtained with UAG and AZP-531, it is hypothesized that administration of AZP-531 will result in decrease of plasma AG levels and/or inhibition of AG bioactivity in patients with PWS. These effects are of particular relevance in patients with PWS as they present higher AG levels as compared to healthy subjects and obese subjects (who have lower AG levels as compared to normal individuals). These effects are expected to translate into decrease in appetite and improvement in hyperphagia and food-related behaviors with potential long-term benefits on obesity and related complications.

1.6 Risk Assessment and Management

Non-clinical findings have indicated that AZP-531 was well tolerated in rats and dogs when administered in repeated doses for 14 days with a wide safety margin. The NOAELs were set at the highest doses tested in each of the species (2 x 18 mg/kg/day and 2 x 5.5 mg/kg/day in rats and dogs, respectively) and represent 100-400-fold the anticipated therapeutic dose range in man (1 to 4 mg/day). In the dog, AZP-531 treatment caused minimal to moderate granulomatous subcutaneous inflammation associated with accumulation of foreign material, likely to be the test item, at some injection sites of a few treated animals from all dose level groups (5 out of 18 animals). No anti-AZP-531 antibodies were noted at the end of the 14-day treatment period in either species.

In the Phase I first-in-man randomized placebo-controlled study (Part A), AZP-531 was administered as a single subcutaneous ascending dose to a total of 33 healthy male volunteers aged 20-47 years old, as follows: 3 subjects received 0.3 µg/kg s.c. and 6 subjects received either 3, 15, 30, 60 or 120 µg/kg s.c. There has been no serious adverse reaction following AZP-531 administration via subcutaneous injection. There have been few possibly related mild adverse events injection site reactions and gastrointestinal symptoms. Mild injection site reactions were observed at 15 µg/kg (two events in 1 subject and pain around injection site in 1 subject), 60 µg/kg (one event in 1 subject), 120 µg/kg (in this group, the dose was split into 2 injections and erythema was noted in 4 patients at one or both injection sites) and 4 events in 2 patients on placebo. No clinically significant findings were noted with respect to haematology, coagulation, blood chemistry, urinalysis, vital signs, ECG and telemetry.

In the subsequent Phase I study part (Part B), a total of 24 overweight / obese subjects otherwise healthy aged 21-61 years old were administered AZP-531 as a daily subcutaneous ascending dose, as follows: 6 subjects/dose group received 3, 15, 30 or 60 μg/kg/day for 14 days. In this study part, AZP-531 was well tolerated. Among definitely or possibly related events, mild injection site reactions were observed in AZP-531-treated groups at 3 and 15 μg/kg (2 events with 3 μg/kg in 2 patients and 3 events at 15 μg/kg in 2 patients) and 1 event in 1 patient on placebo. In addition, mild events of gastro intestinal discomfort (altered bowel habit or loose stools) were observed with AZP-531 in 3 patients (1 event at 15 μg/kg in 1 patient and 2 events at 30 μg/kg in 2 patients) and 1 placebo patient complained of constipation. In addition, one event of headache was observed with AZP-531 15 μg/kg in 1 patient. No clinically significant findings were noted with respect to hematology, coagulation, blood chemistry, urinalysis, vital signs, and ECG. Preliminary analysis of glucose and insulin data suggest that mean blood glucose profile following meal improved after 14 days when AZP-531 was administered at a dose of 60 μg/kg while no change was noted in blood insulin.

AZP-531 is an investigational drug and systemic side-effects that may occur in patients with PWS cannot be reliably predicted. As a general safety measure, patients will be kept at the study center on Day 1, first day of treatment, until end of the day and will return home with accompanying relatives in a light health vehicle.

As granulomatous inflammation was observed locally at the injection sites in the dog toxicology study and injection site reactions possibly related to AZP-531 were noted in a few subjects in Phase I studies, all patients in this study will be monitored for injection site reactions. On Day 1 and Day 14, monitoring of injection site reactions will be performed at the study center by the study personnel. During the rest of the treatment period (ambulatory treatment period), home nurses as well as parents or guardians will be instructed to report events in the patient's diary (the nurse will record events up to 15 minutes post-dose).

Although pre-clinical and clinical studies with AZP-531 have not indicated or revealed any hypoglycemia event to date at the doses tested, a theoretical possibility of hypoglycaemia exists in particular as some patients with PWS may display increased insulin sensitivity (40). Blood glucose will be monitored during the treatment period.

No anti-AZP-531 antibodies were observed in both the rat and dog toxicology studies. In healthy and obese subjects, samples were collected but not analyzed as administration of AZP-531 was well tolerated with no significant drug reactions. Similarly, samples for analysis of antibodies will be collected in this study but not analyzed at first instance.

Safety and efficacy of AZP-531 during pregnancy has not been established. Although fertility is extremely rare in PWS and only a few cases of pregnancy has been reported in the literature (41), all post-menarche female patients who will be included in the study must have a negative pregnancy test on admission. Pregnancy test will also be performed on Day 14. In addition, WCBP, sexually active male subjects, and all male partners of subjects should agree to use adequate methods of birth control throughout the study and for 30 days after the last dose of study drug.

2 Study Objectives

2.1 Primary Objective

The primary objective will be to evaluate the safety and tolerability of one dose of AZP-531 adjusted for bodyweight (2, 3 or 4 mg) administered over 2 weeks as daily subcutaneous injection.

2.2 Secondary Objectives

The secondary objectives will be to evaluate effects of AZP-531 after 2 weeks of administration on fullness/satiety and appetite/prospective food consumption as assessed by Numerical Rating Scales (NRS).

2.3 Exploratory Objectives

- Effects of 2 weeks of administration of AZP-531 will be evaluated on the following:
  - Food-related behavior as assessed by the PWS hyperphagia questionnaire (global score, domain scores, and individual scores for selected questions)
  - Clinical Global and Hyperphagia improvement and/or severity as assessed by the investigator using the Clinical Global Impression (CGI) scale and Clinical Hyperphagia Impression (CHI) scales
  - Blood levels of Acylated Ghrelin (AG) and Unacylated Ghrelin (UAG); AG/UAG ratio
  - Blood levels of glucose, insulin, human Pancreatic Polypeptide (hPP), and IGF-1
  - Food-related anxiety as assessed by Numerical Rating Scale (NRS)
  - Overall behavioral and emotional problems as assessed by the Developmental Behavior Checklist 24 (DBC-P24)
- Effects of AZP-531 will be also evaluated on fullness/satiety, appetite/prospective food consumption, and food-related anxiety as assessed by NRS performed daily during the ambulatory treatment period
- In addition, another exploratory objective of the study will be to evaluate the three NRS included in the study (assessing fullness/satiety, appetite/prospective food and food-related anxiety) by investigating two distinct aspects:
  - Content validity: understanding and relevance from patients' perspective through comprehension tests
  - Measurement properties: test-retest reliability, construct validity and ability to detect change

3 Investigational Plan

3.1 Overall Study Design

The study is a Phase IIa, randomized, double-blind, placebo-controlled, multi-center study evaluating one dose of AZP-531 adjusted for bodyweight (2, 3 or 4 mg) administered once daily for 14 days.

It is planned to involve approximately 7 centers in France and Western Europe. Participating sites will be specialized in the management of patients with PWS. Recruitment period is planned to start in October 2014. Each patient will be treated during 14 days and followed for 14 additional days for safety purposes.

The study schematic is presented below:

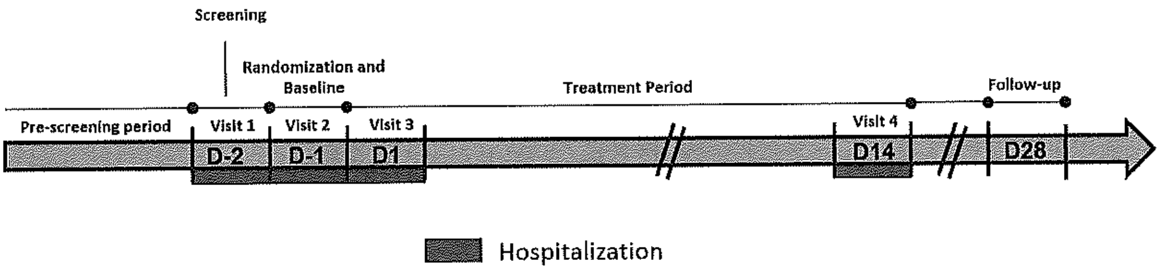

After screening (Day -2), eligible patients will be admitted to the study center and randomized via an Interactive Web Response System (IWRS). Randomization will be 1:1 among treatment arms; stratification will be performed based on genetic subtypes (deletion, non-deletion).

Patients will be evaluated on Day -1 for baseline measurements, on Day 1 following administration of study drug and discharged at the end of the day. They will be admitted to the study center again on Day 13 for evaluation of end of treatment period (Day 14). Patients may stay overnight at a nearby location as long as it is judged by the investigator that this will not interfere with study procedures (e.g. fasting measurements).

Patients will be given a diary in order to record adverse events and NRS data collected during the treatment period at home. During that period, NRS will be administered every morning just before dosing and after breakfast under the supervision of parents and/or guardians as applicable.

On Day -1, Day 1 and Day 14, each subject must consume standardized isocaloric breakfast and lunch (breakfast of same number of calories between Days and lunch of same number of calories between Days). In addition, each of these meals must be comparable in composition between Days (with respect to carbohydrate, lipid and proteins intake). On Day 1 and Day 14, breakfast will be served 30 minutes following dosing.

Follow-up will be performed on Day 28 on the phone. A follow-up visit at the study center may be scheduled within 48 hours, at the investigator's judgment.

Following analysis of unblinded study data and if clinical benefits are observed, AZP-531 will be made available for 14 days for placebo patients. Informed consent should be obtained from legal guardians or from the subjects assisted by their legal representative, as appropriate. Safety data will be collected during this time period.

3.2 Discussion of Study Design

PWS is a rare disease. There is no alternative treatment for hyperphagia, a salient condition of the syndrome that is observed from childhood and is associated with increasing morbidity and mortality over time. The proposed study design is judged acceptable for an initial demonstration of safety, tolerability and efficacy of AZP-531 in this patient population.

3.3 Rationale for Dose Selection

The study doses have been selected based on data obtained so far in clinical studies with UAG and AZP-531.

Early clinical studies with UAG have shown that doses ranging from 45 to 150 µg/kg per day were well tolerated and induced a reduction in blood glucose in T2DM patients. Based on molarity, these doses correspond to approximately 15 to 50 µg/kg per day of AZP-531.

In overweight/obese subjects, AZP-531 administered for 14 days was well tolerated at a daily dose up to 60 µg/kg. At this dose, preliminary analysis of the pharmacodynamic data suggests an improvement of mean blood glucose profile following meal after 14 days of treatment.

For practical reasons, a fixed dose has been selected. The doses correspond to 29 to 67 µg/kg for patients weighting 30 to 140 kg. Patients will receive the following dose of AZP-531 adjusted for body weight.

Body weight	30 to 49 kg	50 to 70 kg	>70 kg
Dose	2 mg	3 mg	4 mg

For patients <30 kg, dose will be adjusted based on actual body weight (60 µg/kg).

3.4 Rationale for the selection of Efficacy Endpoints for Evaluation of Eating Behaviors

To our knowledge, there are no validated clinical endpoints to predict clinical benefit for drugs intended to improve eating behaviors.

In PWS, two types of tools have been designed by academic researchers for use in clinical practice and clinical research: 1) 10- point Visual Analog Scales (VAS) (completed by the patients), some of them include 6 pictorial representations or photographs (7;42), and 2) questionnaires (completed by parents or caregivers).

The scales proposed in this study have been adapted from published VAS evaluating satiety and hunger and described as "how full does your belly feel" and "how much do you think you can eat", respectively. The Sponsor has designed 6-point numeric rating scales (NRS) in order to capture patient feelings with respect to appetite, satiety in addition to anxiety associated with mealtimes.

All the 6 points of the appetite and satiety NRS have been illustrated based on the observation that patients with PWS tend to select points that are associated with images. In order to build universal tools for the population with PWS and in an effort to limit cultural adaptation, cartoons where food items are represented in a neutral way have been added in the appetite instrument, replacing meal photographs as seen in the published tools. Attention has been made in the illustration of food items so that they can be viewed as any of the main daily meals (breakfast, lunch, or dinner). In addition, in order not to project a negative self-image, the satiety NRS has been designed with cartoon characters whose belly is filling instead of swelling more and more.

No illustration of the anxiety NRS has been included at this point. In the 3 NRS, written descriptors have been included but no number has been added on the scale itself as subjects with PWS have been reported to have difficulties rating their feelings on a merely numeric scale.

It is proposed that the appetite, satiety and anxiety NRS are tested in this study in a preliminary way and if appropriate will be further improved, qualified and validated for use as endpoints in pivotal/registration studies. In this perspective, content validity and measurement properties of the proposed NRS will be investigated as exploratory objectives of this study (refer to Section 4.5).

The PWS Hyperphagia questionnaire published by Dykens *et al.* is the most used questionnaire in clinical practice. This questionnaire is a 13-item instrument designed to measure food-related preoccupations and problems in PWS (43). As many questions refer to changes observed over a long time period (weeks and months), it will be used as an exploratory tool in this study.

3.5 Study Population

3.5.1 Inclusion Criteria

Subjects are included in the study if they meet all the following criteria.

- Male and female patients with genetically confirmed diagnosis of Prader-Willi Syndrome using standard DNA methylation test or fluorescent in situ hybridization
- Patients aged 18 years to 50 years old inclusive
- Have evidence of increased appetite or hyperphagia, as judged by the investigator
- Patients on well-balanced controlled diet and on a regular exercise program as recommended in common clinical practice for this patient population
- Women of Child Bearing Potential (WCBP) must have a negative pregnancy test on admission to the study center
- All WCBP, sexually active male subjects, and all partners of subjects should agree to use adequate methods of birth control (diaphragm, condoms with spermicide…) throughout the study and for 30 days after the last dose of study drug
- Adequate renal function, defined as serum creatinine ≤1.5 × Upper Limit of Normal (ULN) and urine protein/creatinine ratio <0.2
- Adequate hepatic function, defined as total bilirubin ≤1.5 × ULN and Aspartate aminotransferase (AST) and Alanine aminotransferase (ALT) levels ≤3 × ULN
- Growth hormone treatment will be permitted if doses have been stable for at least 1 month prior to screening
- Psychotropic treatment will be permitted and should be stable at least 1 month prior to screening
- Any other treatment including thyroid hormones should be stable for at least 1 month prior to screening
- In France, patients must be affiliated to the *sécurité sociale* and under guardianship measures
- Informed consent signed by the legal guardians or by the subject assisted by his/her legal representative, as appropriate

3.5.2 Exclusion Criteria

Subjects are excluded from the study if they meet any of the following criteria:

- History of chronic liver disease, such as cirrhosis or chronic hepatitis due to any cause, or suspected alcohol abuse
- History of acute or chronic pancreatitis
- Type 1 diabetes

- Insulin treatment
- Use of weight loss agents or drugs known to affect appetite (including GLP-1 analogs) within 2 months prior to screening
- Co-morbid condition or disease (such as respiratory disease or psychiatric disorder) diagnosed less than 1 month prior to screening
- Co-morbid condition or disease or abnormal laboratory finding that would in the investigator judgment increase the subject risk to participating in this study and that will not allow the patient to complete the study
- History or presence of gastrointestinal, hepatic or renal disease or any other condition known to interfere with the absorption, distribution, metabolism or excretion of drugs
- Participation in a clinical trial with an investigational agent within 2 months prior to screening
- Clinically significant abnormalities on ECG at screening
- Pregnant or lactating woman
- History of hypersensitivity to drugs with a similar chemical structure or class as AZP-531 (Acylated Ghrelin and Unacylated Ghrelin)
- Unwillingness or inability to follow the procedures outlined in the protocol

3.6 Study Treatment

3.6.1 Study Drug and Clinical Supplies

Study drug will be dispensed by LC2 (Lentilly, France) to the study center in a blinded fashion as treatment kits bearing a kit number. The allocation of study drug kits to patients must only be carried out by the IWRS. For further information on this procedure, please refer to the IWRS user's manual.

Each kit will contain three boxes of treatment (two for the treatment at the clinical center, D1 and D14; and a third for the treatment at home, D2 to D13).

AZP-531 and placebo will be dispensed in same sized 2-mL clear glass vials. Each vial will contain a sterile lyophilized white to off-white powder of comparable aspect at visual inspection (2.2 mg of AZP-531 for the active drug and 5 mg of mannitol for the placebo).

Vial and kit labels will comply with regulatory requirements. Treatment kits will be kept refrigerated (between +2°C and +8°C) either at the study center (locked environment with restricted access) or at patient's home, as applicable, prior to daily reconstitution and use.

Single use vials of sterile 0.9% sodium chloride for injection, alcohol swabs, syringes, biohazard disposal container, ice packs and cooler bags, will also be provided.

3.6.2 Reconstitution and Administration Procedures

Before proceeding to reconstitution, vials of study drug will be removed from the fridge and left at room temperature for a few minutes. Vials will be gently tapped to ensure that all powder is at the bottom.

The study drug will be prepared using one vial for the 2 mg dose and 2 vials for the 3 and 4 mg doses. Vials will be reconstituted with 0.9% sodium chloride for injection, and will be gently swirled and rotated (for at least 15 seconds) to facilitate dissolution of the powder. Based on the dose, an appropriate volume of reconstituted study drug will be injected to patient. Instructions will be provided in a separate document.

Study drug will be administered by a health professional at the study center (Day 1 and Day 14) and at patient's home (from Day 2 to Day 13). Injections will be performed subcutaneously in the abdomen at rotating sites each study morning at approximately the same time. Breakfast will be served 30 minutes following injection.

3.6.3 Randomization Procedures

Patients will be assigned to one of the two treatment groups in a randomized fashion. A computer-generated randomization schedule will be prepared prior to the start of the study. An Interactive Web Response System (IWRS) will be used to assign randomization number and kit number according to the randomization schedule.

Randomization will be 1:1 among treatment arms. Stratification will be performed based on genetic subtypes (deletion vs. non-deletion).

3.6.4 Blinding and Code Breaks

Two sets of sealed envelopes containing the randomization codes will be generated. One set will be provided to the investigator and the other will be kept at Alizé Pharma.

The randomized code may be broken if an emergency situation arises that in the Investigator's opinion requires the knowledge of the code (for example, life threatening situation or necessity to know the product administered to provide with the best medical care). The investigator must do his/her very best to contact the Sponsor at the following number: +33 6 29 21 73 16 before unblinding the code. Date, time, and reason(s) for breaking the code must be recorded by the Investigator on the sealed envelope. Signature of the investigator will be requested.

Randomization data (list and sealed envelopes) are kept strictly confidential, accessible only to authorized persons, until the time of unblinding. It is the responsibility of the Investigator to maintain the blind throughout the study. At the conclusion of the trial, the occurrence of any emergency code breaks will be verified after return of all code break reports and unused drug supplies to the packaging supplier. The drug codes will be broken and made available (unblinding) for data analysis only when the study is completed, the clinical database locked and the protocol violations determined.

The Sponsor reserves the right to break the blind for SAEs which are considered as related to the study drug and unexpected, which could require an expedited report to the regulatory authorities.

3.6.5 Drug Accountability

Each Investigator/designee is responsible for taking an inventory of each shipment of study drug received and comparing it with the accompanying accountability form. The Investigator/designee will verify the accuracy of the information on the form, sign and date it, and return it to Alizé Pharma or its designee.

Study drug must be used only as directed in this protocol. The Investigator/designee must keep accurate written records of all study drug received from Alizé Pharma. Additionally, the Investigator/designee must keep accurate records of the study drug dispensed to patients in this protocol including the number of vials used to prepare patient doses, lot number, date dispensed, and identification number, dose administered, balance forward, and the initials of the person

dispensing the medication. Based on the entries in the site drug accountability forms, it must be possible to reconcile drug product delivered with that used and returned. All study drugs must be accounted for and all discrepancies investigated and documented appropriately.

Study drug stock may not be removed from the investigative site where originally shipped without prior knowledge and consent of Alizé Pharma or their delegated CRO. When authorized, all applicable local, state, and national laws must be adhered to for the transfer.

At the end of the study, all unused vials of study drug will be destroyed by the investigative site or sent to a designated contractor for disposal on behalf of Alizé Pharma, per the instructions at that time. Study drug returned to the Alizé Pharma designated contractors must be counted and verified by site personnel and Alizé Pharma /designee. All certificates of delivery/drug receipts and/or return forms must be signed prior to shipment. Study drug for return must be packed in a tamper-evident manner to ensure integrity by the receiving contractor. All study drug returned must be in accordance with local, state, and national laws and must first be authorized by Alizé Pharma prior to shipment.

3.6.6 Concomitant Medications and Non authorized Medications

Growth hormone treatment will be permitted if doses have been stable for at least 1 month prior to screening.

Psychotropic treatment will be permitted and should be stable at least 1 month prior to screening.

Any other treatment including thyroid hormones should be stable for at least 1 month prior to screening.

All concomitant drugs will be recorded in the Case Report Form, included the ones given for prophylaxis.

Use of weight loss and anorexigenic agents will not be allowed during the study, including GLP-1 analog.

In the interests of patient safety and acceptable standards of care the Investigator will be permitted to prescribe treatment(s) at his discretion if it is considered necessary for the patient's welfare. All treatments must be recorded in the patient eCRFs (medication, dose, treatment duration and indication) throughout the study from providing informed consent until after the final medical examination at the follow-up visit.

3.7 Visit Schedule and Assessments

3.7.1 Visit Schedule

Before the initiation of the study procedures, contact may be performed on the phone or during a routine medical visit. During this contact, the following may be discussed:

- Inclusion/exclusion criteria
- Medical history, disease history, demographics and concomitant medications
- Study objectives, procedures, and informed consent to the legal guardians, as appropriate

This should be planned between 1 to 4 months before screening at the latest, in order to allow sufficient time for obtaining informed consent.

Visit 1: Screening (Day -2)

- Obtain signed informed consent
- Review inclusion/exclusion criteria
- Review medical history, disease history, demographics, IQ (if available) and concomitant medications
- Perform physical examination, obtain vital signs
- Perform urinary pregnancy test
- Perform safety lab tests in fasting condition (haematology, clinical chemistry, coagulation and urinalysis)
- Perform 12-lead ECG
- Administer NRS to patients (see Appendices 3 to 5) for training and acclimation purposes
- Train parents/guardians for administration of NRS to patients during the ambulatory treatment period
- Perform comprehension tests of NRS in study centers located in France (only in France for logistical reasons)

Visit 2: Randomization and Baseline (Day -1)

- Review inclusion/exclusion criteria
- Randomize patients via the IWRS
- Obtain vital signs
- Collect concomitant medication (if applicable)
- Obtain Waist Circumference (WC), Body Weight (BW), fat mass by bio-impedance height, and BMI before breakfast
- Administer NRS at selected time points at breakfast and lunch (before meal, at the end of the meal and 120 min. post-start of meal)
- Record any food item not consumed – No food should be served between breakfast and lunch
- Sample for AG, UAG, insulin, glucose, hPP, before breakfast, and 60 and 180 minutes post-start of breakfast. This later time point sampling must occur before start of lunch. A catheter will be inserted for collection of samples performed on Day-1 and Day 1.
- Sample for AZP-531 antibodies
- The following breakfast time activities must be performed in the following order: NRS, insertion of catheter, sampling for selected measurements, meal.
- Perform the PWS Hyperphagia questionnaire (see Appendix 6) and DBC-P24 (see Appendix 7)
- Perform CHI-S scale (see Appendix 8).
- Record adverse events
- Inform the primary doctor about the inclusion of the patient in the study

Visit 3: Start of Treatment at study center (Day 1)

- Record concomitant medication (if applicable)
- Administer NRS at selected time points at breakfast and lunch (before meal, at the end of the meal and 120 min. post-start of meal)

- Record any food item not consumed – No food should be served between breakfast and lunch
- Sample for AG, UAG, insulin, glucose, hPP, before breakfast, and 60 and 180 minutes post-start of breakfast. This later time point sampling must occur before start of lunch.
- Sample for IGF-1 in fasting condition (before dosing)
- Administer study drug before breakfast
- The following breakfast time activities must be performed in the following order: NRS, sampling for selected measurements, dosing, meal (meal must be taken 30 minutes following dosing).
- Record adverse events
- Dispense study drug and supplies for treatment on Day 2 to 13
- Dispense patient diary and glucometer kit

Ambulatory Treatment Period (Day 2 to 13)

- Record concomitant medication (if applicable)
- Administer NRS every day before dosing and after completion of breakfast
- Obtain fasting blood glucose (before dosing) using glucometer every day and report value in the patient diary
- Administer daily dose of study drug before breakfast (meal must be taken 30 minutes following dosing)
- Record adverse events (the nurse must stay with the patient until 15 minutes after the injection)
- Complete patient diary

Visit 4: End of Treatment Period (Day 14) or Premature Treatment Discontinuation

- Record concomitant medication (if applicable)
- Collect all used and unused study drug vials
- Perform study drug accountability and compliance
- Review patient diary
- Perform physical examination and obtain vital signs
- Obtain WC, BW, fat mass by bio-impedance before breakfast
- Perform safety lab tests under fasting condition (haematology, clinical chemistry, coagulation and urinalysis)
- Perform urinary pregnancy test
- Administer NRS at selected time points at breakfast and lunch (before meal, at the end of the meal and 120 min. post-start of meal)
- Record any food item not consumed – No food should be served between breakfast and lunch
- Sample for AG, UAG, insulin, glucose, hPP, before breakfast and 60 and 180 minutes post-start of breakfast. This later time point sampling must occur before start of lunch. A catheter will be inserted for collection of post-breakfast time points.
- Sample for IGF-1 in fasting conditions (before dosing)
- Sample for AZP-531 antibodies (before dosing)
- Administer study drug before breakfast
- The following breakfast time activities must be performed in the following order: NRS, insertion of catheter, sampling for selected measurements, dosing, meal (meal must be taken 30 minutes following dosing).

- Record adverse events
- Perform the PWS hyperphagia questionnaire and DBC-P24
- Perform CGI-I, CHI-I and CHI-S scales (see Appendix 8).
- The patient must have the same evaluations as those planned at Day 14 in case of premature treatment discontinuation (within 48 hours).

Follow-up (Day 28 ± 3 days) – Phone call and Visit if applicable (Visit 5)

- Patients, parents or guardians (as applicable) interview to assess adverse events
- Patients may be scheduled for a subsequent visit (within 48 hours) for further examination (vital signs, BW and WC as well as physical examination and safety labs under fasting condition), as judged necessary by the investigator.
- The follow-up must be performed even if the patient has prematurely discontinued treatment, 14 days after the last dose of study drug taken (safety follow-up).

Schedule of Assessments is included in Appendix 1

3.7.2 Safety Assessments

This is a study to primarily assess the safety and tolerability of 14 days of daily subcutaneous administration of AZP-531. Safety assessments will include the monitoring and recording of all adverse events and any serious adverse events, the assessment of hematology, blood chemistry, and urinalysis parameters, measurement of vital signs, as well as performance of physical examination. Please refer to Schedule of Assessments in Appendix 1.

Although pre-clinical and clinical studies with AZP-531 have not indicated or revealed any hypoglycemia event to date at the doses tested, a theoretical possibility of hypoglycaemia exists. Patients will be given first dose (Day 1) and last dose (Day 14) of treatment in a hospital environment. In case of symptomatic hypoglycemia, sugar or glucose drinks will be available at the clinical site (if there was no adequate response to oral glucose or the subject is unable to drink the glucose drink, intravenous glucose and intramuscular glucagon will also be available). Patients, parents and guardians, as applicable, will be educated to recognize symptoms of hypoglycemia and will be instructed to take or give sugar if any of these occur during the ambulatory treatment period. Also, fasting blood glucose will be monitored every day using a glucose meter and values will be reported in the Patient diary. Patients, parents and guardians, as applicable, will be instructed to take or give sugar if the fasting glucose value is below 50 mg/dL (2.8 mmol/L) and to repeat glucose check in the next 30 minutes and the next hour. They will be instructed to call the study center if one of the repeat values is below 50 mg/dL (2.8 mmol/L) for further investigation and management.

3.7.2.1 Adverse Events (AE)

Adverse events will be recorded on the Adverse Event case report form throughout the study from providing informed consent until after the final medical examination at the follow-up visit if applicable. Instructions for reporting adverse event are provided in section 5.2.

3.7.2.2 Serious Adverse Events (SAEs)

Information regarding serious adverse events will be collected and recorded on the Serious Adverse Event (SAE) Report Form. Instructions for notification of serious adverse events are provided in section 5.3.

3.7.2.3 Safety Laboratory Evaluations

Except for measurement of AZP-531 antibodies, safety laboratory evaluations will be analysed by the site's local laboratory.

- Hematology

Hemoglobin, hematocrit, platelet count, WBC count, RBC count, and WBC differential.

- Blood chemistry

AST, ALT, GGT, alkaline phosphatase, total bilirubin, Creatine PhosphoKinase (CPK), Lactate DeHydrogenase (LDH), creatinine, urea, amylase, lipase, uric acid, glucose, cholesterol, LDL, HDL, triglycerides, sodium, potassium, calcium, chloride, protein, albumin.

- Coagulation

Prothrombine time, activated partial thromboplastin time.

- Urinalysis

Specific gravity, pH, leukocytes, protein, glucose, ketones, bilirubin, creatinine, RBCs, nitrites, urobilinogen.

- AZP-531 antibodies

3.7.2.4 Vital Signs

Respiration rate, pulse rate and sitting blood pressure after 5 minutes of rest.

3.7.2.5 Physical Examination

The examination of the following will be performed: eyes, ears, nose and throat, heart, peripheral vasculature, lungs, muskuloskeletal system, abdomen, neurologic function, endocrine system, genito-urinary system, skin and lymph nodes.

Significant findings that are present prior to the start of the study drug must be included in the Relevant Medical History case report form or Current Medical Conditions case report form. Significant findings made after the start of the study drug which meet the definition of an AE must be recorded in the Adverse Event case report form.

3.7.2.6 Body Weight

Subjects will be weighted clothed (underwear) without shoes using the same balance.

3.7.3 Efficacy Assessments

Patients will be asked to rate fullness/satiety, appetite/prospective food consumption using NRS that have been specifically designed for the PWS patient population.

Completion of these scales will be performed under the supervision of a health professional (assessments at the study center) at selected time points on Day -1, Day 1, and Day 14, as follows: before breakfast (and before dosing when applicable), at the end of breakfast and lunch, and 120 min. post-start of breakfast and lunch. Please refer to Schedule of Assessments in Appendix 1.

3.7.4 Exploratory Efficacy Assessments

Schedule of assessment is provided under Appendix 1.

3.7.4.1 PWS hyperphagia questionnaire

This questionnaire is a 13-item instrument designed to measure food-related preoccupations and problems in PWS (43). It will be completed by a health professional on Day-1 and Day 14 following interview of patients, parents or guardians as applicable.

3.7.4.2 Clinical Global Impression (CGI) scale and Clinical Hyperphagia Impression (CHI) scales

Clinical Global Impression scales have been developed by Guy W. in 1976 as research tools for clinical practice and have been since extensively used in clinical trials. They evaluate either severity of illness (CGI-S) or clinical global improvement (CGI-I) on a 7-point scale (44). Scales for severity of hyperphagia (CHI-S) and hyperphagia improvement (CHI-I) have been adapted from these tools. All these scales will be completed by the investigator.

3.7.4.3 Hormonal and other laboratory assessments

Central laboratories will be used for assays described in this section. Names, addresses and information concerning sample collections and shipment, as well as manuals describing processing of samples will be provided separately.

- Acylated Ghrelin (AG) and Unacylated Ghrelin (UAG)
- human Pancreatic Polypeptide (hPP)
- IGF-1
- Plasma glucose and serum insulin

3.7.4.4 Food-related anxiety

Assessment will be performed using a NRS on Day -1, Day 1 and 14 at the same time as fullness/satiety, appetite/prospective food consumption assessments; this scale will be completed by the patient under the supervision of a health professional.

3.7.4.5 Developmental Behavior Checklist

DBC is a questionnaire that has been designed for the assessment of behavioral and emotional problems of patients with developmental and intellectual disabilities. DBC-P24 is a short form of DBC (24 questions) that has been initially developed for children and adolescents. As questions also apply to adult patients, this questionnaire will be used for the whole study population.

This questionnaire will be completed by a health professional following interview of patients, parents or guardians as applicable.

3.7.4.6 Additional assessments of fullness/satiety, appetite/prospective food consumption and food-related anxiety

These assessments will be performed using NRS every day during the ambulatory treatment period before and after breakfast, under the supervision of parents or guardians as applicable.

3.7.4.7 Comprehension tests of NRS

Comprehension test is a qualitative research method to evaluate the content validity of a tool, i.e. its understanding and relevance (45;46). This approach assesses whether items (item stem and response options), questionnaire instructions, and recall period are understood by patients as intended by the item designer. This is evaluated by analyzing the process of answering questionnaire items, identifying how and when an item does not achieve its objective.

For these comprehension tests, patients will be asked structured questions following a standardized interview guide, which describes comprehensively the full interview process (instruction to the interviewers, specific list of questions to ask to the patient, etc.). This interview guide is provided in Appendix 9.

Patient's interview will be conducted by a healthcare professional, who will have been trained to the conduct of comprehension tests at the initiation of the study. Comprehension tests will be performed on Day-2 following the training and acclimation session with NRS. Interviews will be audio-recorded and transcribed word-by-word for analysis purpose.

For logistical reasons, these comprehension tests will be conducted with 20 patients recruited in selected centers in France.

3.8 Criteria for Discontinuation

Subjects may discontinue participation in this clinical study for the following reasons:

- Withdrawn consent
- Adverse event
- Lack of compliance to protocol requirements and/or procedures
- Lost to follow-up
- Administrative deviations which preclude satisfactory completion of protocol assessments
- Death

Before interrupting or discontinuing study drug, the investigator must contact the Sponsor or designee (except in the case of an emergency) to discuss the proposed reason. It will be documented whether or not each subject completed the clinical study. If any subject's study treatment or observations were discontinued, the reason will be recorded in the patient's file and in the case report form.

In case of premature treatment discontinuation, the patient must be assessed for a final evaluation (assessments planned at Day 14) and contacted for the follow-up assessment 14 days after the last dose of study drug taken (safety follow-up).

4 Statistical Analyses

4.1 Determination of Sample Size

4.1.1 Interim Analysis

Up to 20 patients aged 18 to 50 years old (with a minimum of 8 patients treated within each of the AZP-531 and Placebo group) who have completed the treatment period (Day 14) will be part of the interim analysis. An unblinded statistician will follow-up the study completion of

the patients to make sure that a minimum of 8 patients treated within each group will be included. Eight (8) treated patients, corresponding to 40% of the patients in the AZP-531 group, are considered sufficient to have an overview of the safety results and identify major outstanding safety issue.

4.1.2 Final Analysis

Approximately 40 evaluable subjects will be enrolled in the study. A number of 20 patients per group is considered appropriate for initial evaluation of safety and preliminary efficacy of AZP-531 in this patient population. With 20 patients per arm, the probability to detect at least one adverse event per arm whose actual frequency is ≥ 8%, is of more than 80%. Thus, adverse events which happen with an incidence of 8% or more will most likely be captured and described in the safety evaluation of this study. The exact 80% confidence intervals (Clopper-Pearson), will be [0.5%; 18.1%], [2.7%; 24.5%] and [5.6%; 30.4%] if 1, 2 and 3 AEs out of 20 patients are observed, respectively. Moreover, with this sample size, and based on a preliminary evaluation of fullness/satiety Visual Analog Scales (VAS) data published in the literature (7;42), we will have about 80% power to detect a difference of 1 unit score on the VAS scale, assuming a standard-deviation of 1.26 and a type I error of no more than 10%, two-sided.

It is to be noted that the published VAS included 10 unit scores while the NRS tools used in this study include 6 unit scores (from 0 to 5). Since the range of the scale is smaller, the variances might be lower than the ones observed in the literature. We thus believe that the sample size calculated for this protocol is conservative.

4.2 Population Sets

4.2.1 Full Analysis Set

The Full Analysis (FA) set will include all randomized patients who received at least one dose of the study medication. The initial randomization will be preserved for this analysis set in order to comply with the Intent-To-Treat (ITT) principle.

The FA set will represent the primary analysis population to evaluate the treatment groups in term of effectiveness as defined in section 4.4.3 and 4.4.4.

4.2.2 Safety Set

The safety set will include all randomized patients who received at least one dose of the study medication. The treatment actually received by the patient will be used in this set.

4.2.3 Per Protocol Set

The Per Protocol set (PP) will include all randomized patients who:

- Completed the 14 days of treatment.
- Have non-missing observation at baseline (Day -1) and Day 14 for at least one of the fullness/satiety or appetite/prospective food consumption, as assessed by Numeric Rating Scales (NRS).
- Have no major protocol violations including the violation of inclusion/non inclusion criteria.

The treatment actually received by the patient will be used in this set. Prior to database lock, major protocol violations will be defined and reviewed by Alizé Pharma and the CRO in charge

of the study follow-up in a blinded fashion. Patients with major protocol violations will be excluded from the PP population.

The PP set will be used as secondary set to support the FA analyses.

4.3 Missing Observations

No imputation of missing observations will be done and all analyses will be done on the Observed Case (OC).

4.4 Description of Statistical Methods

4.4.1 Demographic and Baseline Characteristics

Demographic and baseline characteristics will be summarized by treatment group using descriptive statistics.

Analyses of demographic and baseline characteristics will be performed on the FA, Safety and PP sets.

4.4.2 Evaluation of Safety

The evaluation of safety will be based on the safety population. The data collected will be presented in listings, summary tables, and graphs to give an overview of the safety findings.

4.4.2.1 Adverse events

AEs will be collected from the signature of informed consent. AEs will be classified as treatment-emergent AEs (TEAEs) or pre-treatment AEs. An AE with an unknown/unreported onset date will also be counted as a treatment-emergent AE. Unless otherwise stated, all references to AEs in this study protocol refer to treatment-emergent AEs. An increase of intensity or frequency of a pre-treatment AE will also be considered as a TEAE.

The number and percentage of subjects, who experience non-serious AEs, as well as serious AEs, will be presented by System Organ Class (SOC) and by Preferred Term (PT) within SOC for each treatment group. Non-serious and serious AEs will be similarly presented by severity, by relationship to study drug, and by outcome of events. Moreover, the number of AEs will be presented (in addition to the number and percentage of subjects who experience AEs). Non-serious and serious AEs will be presented for each group. The total number of non-serious or serious AEs, as well as the total number of subjects with a non-serious or serious AE, will also be presented.

A subject experiencing the same treatment-emergent AE multiple times will be counted only once for the corresponding PT. Similarly, if a subject experiences multiple AEs within the same SOC, the subject will be counted only once for that SOC. If a subject experiences more than one AE within different severity or relationship categories within the same SOC/PT, only the worst case (worst severity and related AE) will be reported. AEs will be sorted alphabetically by SOC and within each SOC the PT will be presented by decreasing order of total frequency. AEs will be coded to SOC and PT using the Medical Dictionary for Regulatory Activities (MedDRA).

A listing of SAEs and discontinuations due to AEs will be presented. Subject deaths will be listed separately. SAEs will be described by case narratives. All AEs will be presented by investigative site and by treatment group in a listing, which will include the subject identifier, PT, reported term, severity, seriousness, action taken, outcome, relationship, date of onset, duration, end date, and study treatment at the time of the event of most recent study treatment taken. Separate listings will be provided for pre-treatment and post-treatment events.

4.4.2.2 Safety Laboratory Evaluations

Descriptive statistics (n, mean, standard deviation, median, and range) for each clinical laboratory test will be presented by treatment group and available visit. Change from baseline to each available post-dose visit will also be summarized.

According to the laboratory normal ranges, laboratory test results will be categorized as low (< lower normal limit), normal (within normal range), and high (> upper normal limit). Shift tables comparing the distributions of these three categories at baseline versus each available post-dose visit will be presented by treatment group for key safety laboratory evaluations. Moreover, a summary of newly occurring or notable worsening laboratory abnormalities will be presented. The criteria to identify these laboratory abnormalities will be defined in the statistical analysis plan.

4.4.2.3 Vital Signs, Waist Circumference (WC), Weight, Fat mass, Height and BMI

Descriptive statistics (n, mean, standard deviation, median, and range) will be presented for data related to vital signs, WC, weight, fat mass, height and BMI. Change from baseline values will also be presented for each post-baseline measurement (with the exception of height).

4.4.2.4 Physical Exam

Physical Exam will be summarized by visit in terms of n (%) of patients with normal/abnormal results per body system and treatment. Moreover, shift tables (from normal to abnormal and abnormal to normal) will be presented at Visit 4 (Day 14).

4.4.3 Evaluation of Efficacy

Analyses of efficacy will be performed on FA and PP sets.

The NRS: fullness/satiety and appetite/prospective food consumption will be presented descriptively by time (before meal, just after meal, 120 minutes after meal), meal (breakfast, lunch), visit (Day -1, Day 1 and Day 14) and treatment.

<u>Analysis on NRS Score: Within Day</u>

A repeated analysis of variance will be performed on the NRS score at Day 14. The model will include NRS score at baseline (Day -1) as time-dependent covariate, Time, Meal, the stratification variables (genetic subtype), and treatment as fixed effects. The appropriate variance-covariance matrix will be used to model the NRS scores through time and meal within one subject. Key interaction terms such as Time-by-Meal-by-Treatment, Time-by-Treatment,

Meal-by-Treatment as well as interactions between the stratification variable and treatment (subgroup analyses) will also be investigated.

The same model will be performed on NRS score at Day 1 as supportive analysis.

Analysis on NRS Score: Between Days

Moreover, a repeated analysis of variance will be performed on the NRS score at Day -1, Day 1 and Day 14. The model will include Day, Time, Meal, the stratification variable genetic subtype, and treatment as fixed effects. The appropriate variance-covariance matrix will be used to model the NRS scores through day, time and meal within one subject. Key interaction terms such as, Day-by-Treatment, Day-by-Time as well as interactions between the stratification variables and treatment (subgroup analyses) will also be investigated. The comparison of interest will be: Day 14 vs. Day -1, Day 1 vs. Day -1, and Day 14 vs. Day 1.

Analysis on NRS Score assessed during the Ambulatory Treatment Period (Day 2 to Day 13)

In addition, a repeated analysis of variance will be performed on the NRS scores from the NRS completed by the patients. The model will include the appropriate NRS score at baseline (Day -1) as time-dependent covariate, Day, Time (before or after breakfast), the stratification variable genetic subtype, and treatment as fixed effects. The appropriate variance-covariance matrix will be used to model the NRS scores through day and time within one subject. Key interaction terms such as, Day-by-Treatment, Day-by-Time as well as interactions between the stratification variable and treatment (subgroup analyses) will also be investigated.

All the analyses will be performed for fullness/satiety, and appetite/prospective food consumption separately. All analyses will be performed on FA and PP sets.

4.4.4 Exploratory Evaluations

The results described below will be presented on both the FA and PP sets.

4.4.4.1 Food-related behavior using the PWS hyperphagia questionnaire (global score, domain scores, and individual scores for selected questions)

The PWS hyperphagia questionnaire global, domain and individual scores for selected questions will be presented descriptively by visit (Day -1 and Day 14) and treatment.

Moreover, an analysis of covariance will be performed on the Score (global, by domain, and individual score) at Day 14. The model will include the score at baseline (Day -1) as covariate and the stratification variable (genetic subtype) and treatment as fixed effects. Interactions between the stratification variables and treatment will also be investigated in order to present some subgroup analyses.

4.4.4.2 Clinical Global Impression (CGI) scale and Clinical Hyperphagia Impression (CHI) scales

The CGI and CHI results will be presented descriptively at Day -1 (CHI-S) and Day 14 (CGI-I, CHI-I and CHI-S), per treatment.

CGI-I and CHI-I

An analysis of variance will be performed on CGI-I and CHI-I at Day 14. The model will include the treatment and the stratification variable genetic subtype as fixed effects. Interactions

between the stratification variable and treatment will also be investigated in order to present some subgroup analyses.

CHI-S

An analysis of covariance will be performed on the CHI-S score at Day 14. The model will include the score at baseline (Day -1) as covariate and the stratification variable genetic subtype and treatment as fixed effects. Interactions between the stratification variable and treatment will also be investigated in order to present some subgroup analyses.

4.4.4.3 Blood levels of AG, UAG, hPP, glucose and insulin

The blood levels for AG, UAG, hPP, glucose, insulin and AG/UAG ratio will be presented descriptively by time (before breakfast, 60 and 180 minutes post-start of breakfast), by visit (Day -1, Day 1 and Day 14) and treatment.

Moreover, a repeated analysis of variance will be performed on the blood levels AG, UAG, hPP, glucose and insulin. The model will include the blood level at baseline (Day -1) as a time dependent covariate, the stratification variable genetic subtype, Time (before breakfast, 60 and 180 minutes post-start of breakfast) Day (1 or 14) and treatment as fixed effects. The appropriate variance-covariance matrix will be used to model the blood levels through day and time within one subject. Key interaction terms such as, Day-by-Treatment, Day-by-Time as well as interactions between the stratification variables and treatment (subgroup analyses) will also be investigated.

4.4.4.4 Blood levels of IGF-1

The blood levels of IGF-1 will be presented descriptively by visit (Day 1 and Day 14) and treatment.

Moreover, an analysis of covariance will be performed on the blood levels of IGF-1 at Day 14. The model will include the blood level at baseline (Day 1) as covariate, and the stratification variable genetic subtype, and treatment as fixed effects. Subject will be included as a random effect. Interactions between the stratification variables and treatment (subgroup analyses) will also be investigated.

4.4.4.5 Food-related anxiety

Food-related anxiety, as assessed by NRS, will be analyzed using the same approach as described in Section 4.4.3.

4.4.4.6 Developmental Behavior Checklist (DBC-P24)

The DBC-P24 global score and individual scores for selected questions will be presented descriptively by visit (Day -1 and Day 14) and treatment.

For all statistical analyses described above in sections 4.4.3 and 4.4.4, assumptions of normality and homogeneity of variance will be examined. If these assumptions are not met, transformation of the dependent variable or non-parametric approaches will be considered.

4.5 Evaluation of the NRS

4.5.1 Exploration of NRS content validity: qualitative methods

The content validity of the NRS will be investigated by exploring the understanding and relevance of the NRS to patients of the target population using data collected during comprehension tests.

Twenty patients recruited at selected sites in France will be interviewed to document their understanding of the three NRS and the relevance of these NRS to them. Conducting 20 interviews will be sufficient to capture comprehensively the perception of the patients towards the three NRS.

Patients' verbatim and interviewers' notes related to each element of the NRS evaluated during the comprehension tests (questions, answer choices, pictures) will be documented and analyzed. In a first step, the individual perception of each patient on the elements of the NRS (questions, answer choices, pictures) will be qualitatively evaluated and documented in an evaluation grid. In a second step, the perception of all interviewed patients will be compiled. Finally, potential modifications of each element of the NRS may be suggested based on patients' feedback for future studies.

4.5.2 Preliminary assessment of the measurement properties of the NRS

The measurement properties of the three NRS will be assessed using the data collected during the study. The following measurement properties will be assessed:

- Test-retest reliability: assessment of the stability of NRS over time when no change is expected in the concept of interest
- Construct validity: generation of evidence that the NRS relate with other parameters (PWS hyperphagia questionnaire scores, assessment pre/post meal, etc.) in line with a priori logical hypotheses
- Ability to detect change: generation of evidence that the NRS can identify differences in scores over time in individuals who have changed with respect to the concept of interest

The analyses aiming to document these measurement properties of the NRS will be conducted in the FA set regardless of the treatment group. They will be fully described in a stand-alone specific statistical analysis plan.

4.6 Interim Data Analysis

The DMC will review the blinded safety data of up to 20 patients (with a minimum of 8 patients treated within each of the AZP-531 and Placebo group)..

5 Safety-Related Procedures

5.1 Definitions

5.1.1 Adverse Event

All adverse events which occur during the study period from the signature of the Informed Consent until the final study evaluation (Follow-up visit performed on Day 28 or earlier in case

of premature study discontinuation) will be recorded on the Adverse Event form of the electronic Case Report Form (eCRF).

An Adverse Event (AE) is defined as any untoward medical occurrence in a patient or clinical investigation subject administered with a pharmaceutical product and which does not necessarily have to be related with this treatment.

An AE can therefore be any clinically significant sign (including an abnormal laboratory finding, for example), symptom or disease temporally associated with the use of a medicinal product, whether or not considered related to the medicinal product.

In clinical studies an AE can include an undesirable medical condition occurring at any time, including screening period, even if no study treatment has been administered.

Any overdose of study drug (whether or not it is incidental) will be recorded as AE only if it leads to an AE.

An abnormal laboratory test value should be reported as an AE if any of the following applies:

- It is accompanied by clinical symptoms or is considered as medically significant by the investigator
- It results in a change in the study treatment schedule of administration (for example, delay in administration, temporary or definitive discontinuation)
- It requires an intervention, a change in concomitant treatments, or a diagnostic evaluation in order to determine the risk for the patient

An Adverse Drug Reaction (ADR) is any untoward and unintended response in a subject to a medicinal product which is related to any dose administered to that subject.

An Unexpected Adverse Drug Reaction is an ADR, the nature, severity or outcome of which is not consistent with the applicable product information.

In this study, the reference document for assessing the expectedness of ADR will be the current version of the Investigator's Brochure.

5.1.2 Serious Adverse Event and Suspected Unexpected Serious Adverse Reactions

Information regarding serious adverse events will be collected and recorded on the Serious Adverse Event (SAE) Report Form. A Serious Adverse Event is defined as any untoward medical occurrence that occurs at any dose and:

- results in death,
- is life-threatening,
- requires inpatient hospitalization or prolongation of existing hospitalization,
- results in persistent or significant disability / incapacity, or
- is a congenital anomaly/birth defect in the offspring of a subject who received study drug.

Important medical events that may not be immediately life-threatening or result in death or hospitalization may be considered an SAE when, based upon appropriate medical judgment, they may jeopardize the subject prognosis or may require medical or surgical intervention to prevent one of the outcomes listed in the definition above. Examples of such medical events include allergic bronchospasm requiring intensive treatment in an emergency room or at home, blood dyscrasias, or convulsions that do not result in patient hospitalization, or the development of drug dependency or drug abuse.

Suspected Unexpected Serious Adverse Reactions (SUSAR) are suspected AEs that are considered related to the study drug and are both unexpected and serious.

SUSAR are subject to expedited reporting to the Competent Authorities and Ethics Committees.

5.2 Reporting of Adverse Events

All AEs either reported by patients/parents/guardians or noted by the investigator (during physical exam, review of safety lab. etc) must be fully recorded in the patient's source documents throughout the entire study and will be transcribed into the patient's eCRF, whether or not they are considered to be related to the study drug.

Each AE should be described in detail: nature of the event (a sign, a symptom or a diagnosis, preferably), onset time and date, offset time and date (if applicable), seriousness, intensity, relationship to the investigational product, action taken including corrective therapy, and outcome.

Adverse event should be followed until the event or its sequelae are resolved or stabilized at a level acceptable to the investigator. This can sometimes mean that the follow-up continues after the patient study termination. In the event of a patient not returning to the study site, the outcome of this event will be recorded as "unknown".

5.3 Reporting of Serious Adverse Events and Suspected Unexpected Serious Adverse Reactions

5.3.1 Initial SAE reporting

Serious adverse events occurring during the study period from the signature of the Informed Consent until the final study evaluation (Follow-up visit performed on Day 28 or earlier in case of premature study discontinuation) have to be reported by the investigator **immediately or no later than 24 hours** from the date of first awareness to :

ICTA PM Pharmacovigilance Department

by fax: +800 53 40 53 40 or by email: pharmacovigilance@icta.fr

The Investigator (principal investigator or any other investigator designed by the principal investigator as authorized to notify safety issues) will be requested to complete and sign a separate SAE report form in addition to the information on the eCRF. The SAE report will be generated from a specific module in the eCRF.

All available information concerning the SAE (anonymous copies of laboratory results, other exams, hospitalization reports, autopsy report and other appropriate documents) will be transmitted with the SAE report form.

Further to the notification of a SAE, additional data related to the SAE could be asked by the Sponsor or its representative (by fax, phone, mail or visit). The investigator must answer to these requests for additional information.

ICTA PM Pharmacovigilance Department will notify the Sponsor immediately following awareness. Notification should be performed by phone +33 6 29 21 73 16 and e-mail to drugsafety@alz-pharma.com. ICTA PM will be in charge of reporting the SUSARs to the Competent Authorities and Ethics Committees (EC) based on the information transmitted by the investigator.

A suspected unexpected serious adverse reaction (SUSAR), which is fatal or life-threatening must be reported to the Competent Authorities and EC immediately (within 7 days) after the Sponsor becomes aware of the event. The local regulatory requirements for expedited reporting to competent authorities and EC of each involved country will be described in the safety management plan.

A SUSAR, which is not fatal or life-threatening, must be reported to the Competent Authorities and EC as soon as possible (within 15 days) after the Sponsor becomes aware of the event.

The full requirements of the ICH Guideline for Clinical Safety Data Management, Definitions and Standards for Expedited Reporting, Topic E2, will be adhered to. The study will comply with all local regulatory requirements.

In some cases, some events can be considered as not to be reported as a SAE. These are:

- Hospitalization for elective surgery or treatment / procedures which was planned before signature of the informed consent for a pre-existing condition/disease that has not worsened since the start of the study
- Hospitalization for comfort or social reasons
- Urgent consultation at the hospital for an outpatient (without overnight hospitalization) unless it meets one of the seriousness criteria described in paragraph 5.1.2

5.3.2 SAE follow-up

For any new information related to an already notified SAE, the investigator will have to complete a SAE Follow-up report which will be transmitted by fax/email, within 24 hours, to

ICTA Pharmacovigilance Department

by fax: +800 53 40 53 40 or by email: pharmacovigilance@icta.fr

accompanied with the applicable laboratory results, other exams and/or the hospitalization reports (anonymous copies), as applicable.

The reporting of the follow-up information will be done in the same manner as initial SAE reports.

The SAE must be followed until resolution or stabilization (in case of sequelae) of the event or until patient's death. This can sometimes mean that the follow-up continues after study termination.

5.3.3 SAE occurring after the study

All SAE occurring at any time after the end of the study, likely to be related to the study drug or to a study procedure, according to the investigator, must be notified according to the process of reporting described above.

5.4 Categorization of Adverse Events

The intensity of an AE will be categorized as follows:

Mild:	Mild events are those, which are easily tolerated with no disruption of normal daily activity.

Moderate:	Moderate events are those, which cause sufficient discomfort to interfere with daily activity.
Severe:	Severe events are those, which incapacitate and prevent usual activity.

The term "severe" is used to describe the intensity (severity) of a specific event. Note that it is not the same as "serious", which is based on patient/event outcome or action criteria.

5.5 Causal Relationship Assessment

Causal relationship assessment to the investigational products is required for purposes of reporting AEs. To promote consistency, the following guidelines should be taken into considerations along with good clinical and scientific judgment when determining the relationship of the investigational products to an AE:

Definitely related:	A clinical event, including laboratory test abnormality, occurring in a plausible time relationship to the investigational product administration, and which concurrent disease or other drugs or chemicals cannot explain. The response to withdrawal of the investigational product should be clinically plausible.
Possibly related:	A clinical event, including laboratory test abnormality, with a reasonable time sequence to the investigational product administration but which could also be explained by concurrent disease or other drugs or chemicals. Information on the investigational product withdrawal may be lacking or unclear.
Unlikely related:	A clinical event, including laboratory test abnormality, with little or no temporal relationship to the investigational product administration, and which other drugs, chemicals or underlying disease provide plausible explanations.
Not related:	A clinical event, including laboratory test abnormality that has no temporal relationship to the investigational product or has more likely alternative aetiology.

5.6 Reporting of pregnancy

Pregnancy is exceptionally observed in PWS. If a patient becomes pregnant during the study treatment period or within 14 days after the last dose of study drug the pregnancy will have to be notified. If the pregnancy occurs during the treatment period, the study drug should be discontinued if this can be done safely.

The pregnancies must be reported by the investigator like SAEs, **within the 24 hours** of awareness. The reporting of pregnancy will be done according to the procedure used for SAEs reporting with the completion of a SAE report.

The Sponsor or its representative must follow each pregnancy to term. The pregnancy outcome should be reported to the Sponsor or its representative by the investigator.

6 Ethics and Good Clinical Practice

This study must be carried out in compliance with the protocol and in accordance with the following documents:

- ICH Harmonized Tripartite Guidelines for Good Clinical Practice CPMP/ICH/135/95, 1996.
- ICH E11 Clinical Investigation of Medicinal Products in the Paediatric Population 2001
- Directive 91/507/EEC, The Rules Governing Medical Products in the European Community
- Directive 2001/20//EC, The laws, regulations and administrative provisions of the Member states relating to the implementation of good clinical practice in the conduct of clinical trials on medicinal products for human use
- Detailed guidance on the request to the competent authorities for authorization of a clinical trial on a medicinal product for human use, the notification of substantial amendments and the declaration of the end of the trial (CT1)
- Directive 2005/28/EC, laying down principles and detailed guidelines for good clinical practice as regards investigational medicinal products for human use, as well as the requirements for authorisation of the manufacturing or importation of such products
- Declaration of Helsinki, concerning medical research in humans (Recommendations - Guiding Physicians in Biomedical Research Involving Human Subjects, Helsinki 1964, amended Tokyo 1975, Venice 1983, Hong Kong 1989, Republic of South Africa 1996, and Scotland 2000, Washington DC 2002, Tokyo, 2004, Seoul 2008 and Fortaleza 2013)

A copy of the Declaration of Helsinki is provided in Appendix 2.

6.1 Institutional Review Board / Ethics Committee

Before implementing this study, the protocol, the proposed informed consent form and other information to subjects, must be reviewed by a properly constituted Institutional Review Board / Ethics Committee (IRB/EC). A signed and dated statement that the protocol and informed consent have been approved by the IRB/EC must be given to Alizé Pharma or designee before study initiation. The name and occupation of the chairman and the members of the IRB/EC must be supplied to Alizé Pharma or designee. Any amendments to the protocol, other than administrative ones, must be approved by this committee.

6.2 Informed consent

According to local requirements and GCP, the investigator must explain orally to each subject and/or legally authorized representative the nature of the study, its purpose, the procedures involved, the expected duration, the potential risks and benefits involved and any discomfort or constraint it may entail. Each subject must be informed that participation in the study is voluntary and that he/she may withdraw from the study at any time and that withdrawal of consent will not affect his/her subsequent medical treatment or relationship with the treating physician.

An information leaflet written in lay language will be provided to subjects represented by a legal guardian to explain the study. From an educational point of view, this information leaflet will be asked to be dated and signed by the subject insofar as possible. In all cases, the first name and name will be asked to be mentioned by the subject.

The informed consent should be given by means of a standard written statement, written in non-technical language. The legally authorized representative or the patient assisted by his/her legal representative, as appropriate should read and consider the statement before signing and dating it, and should be given a copy of the signed document. No subject can have a study-related procedure performed before informed consent has been obtained. The assistance of the legally acceptable representative will be documented in the patient medical file.

The informed consent form is part of the protocol and must be submitted for IRB/EC approval. Alizé Pharma or designee supplies a proposed informed consent form, which complies with regulatory requirements and is considered appropriate for the study. Any changes to the proposed consent form suggested by the Investigator must be agreed to by Alizé Pharma or designee before submission to the IRB/EC and a copy of the approval version must be provided to Alizé Pharma or designee after IRB/EC approval. In addition, any change that may affect patients' participation and/or safety will necessitate patients to re-consent.

The informed consent will be available in triplicate. The first copy is for the subject, the second one for the investigator and the third one for the Sponsor. This latter copy will be collected for filing by the Sponsor, under sealed envelope.

The subjects and parents and/or legally authorized representative, will have a patient card with them to show to any other physician met during the study in order to draw her/his attention on a clinical study participation.

7 Data Monitoring Committee

A Data Monitoring Committee (DMC) will review the safety data of the study on a regular basis. An interim analysis (blinded data) will be performed on the first patients (up to 20 patients with a minimum of 8 patients treated within each of the AZP-531 and Placebo group).

The Data Monitoring Committee (DMC) will be composed of independent experts external to the study including at least one clinician knowledgeable in PWS (pediatrician) and one Pharmacovigilance specialist.

A charter will define roles and responsibilities of DMC members.

8 Administrative Procedures

8.1 Changes to the Protocol

Any change or addition to this protocol requires a written protocol amendment that must be approved by Alizé Pharma and the Coordinating Principal Investigator before implementation.

Amendments significantly affecting the safety of subjects, the scope of the investigation or the scientific quality of the study, require additional approval by the IRB/EC of all centers, and, when applicable, by the regulatory authority. A copy of the written approval of the IRB/EC, which becomes part of the protocol, must be given to Alizé Pharma.

These requirements for approval should in no way prevent any immediate action from being taken by the investigator or by Alizé Pharma in the interest of preserving the safety of all subjects included in the trial.

Amendments affecting only administrative aspects of the study do not require formal protocol amendments or IRB/EC approval but the IRB/EC of each center must be kept informed of such administrative changes (e.g. correcting for typographical errors, rewording for clarity, changes in study personnel).

8.2 Quality

This study will be conducted in compliance with the protocol, current GCP rules and the applicable regulatory requirements.

Quality Control comprises the operational techniques and activities undertaken within the quality assurance system to verify that the requirements for quality of the trial-related activities have been fulfilled.

Monitoring is the act of overseeing the progress of a clinical trial, and of ensuring that it is conducted, recorded, and reported in accordance with the protocol, Standard Operating Procedures (SOPs), GCP and the applicable regulatory requirement(s). As part of the supervision of the study progress, the Sponsor personnel or the CRO may, on request, accompany the monitor on visits to the study site. The investigator and the study investigator collaborators commit to cooperate with the monitor to resolve any problems, corrections, or possible misunderstandings concerning the findings or protocol violations detected in the course of these monitoring visits.

Protocol violations will be sorted in significant and non-significant violations before the blind data review. All significant violations will be defined with the Sponsor before the first patient is included in the study. A significant violation is any violation identified during monitoring that requires immediate information to the Sponsor for possible action (for example, withdrawal of the patient from the study).

During the data review and before unblinding, protocol violations will be sorted in major and minor violations. A major violation is any violation having an impact on the classification of the population sets (for example, impacting the main evaluation criteria).

8.3 Monitoring Procedures

Before study initiation, an Alizé Pharma representative will review the protocol with the investigators and their staff. During the study the Alizé Pharma monitor or designee will visit the sites regularly, to check the completeness of patient records, the accuracy of entries in the eCRFs, the adherence to the protocol and to GCP, the progress of enrollment, and also to ensure that study medication is being stored, dispensed and accounted for according to specifications. The investigator and key trial personnel must be available to assist the Alizé Pharma monitor or designee during these visits.

The investigator must give the monitor access to relevant hospital or clinical records, to confirm their consistency with the eCRF entries. No information in these records about the identity of the subjects will leave the study center. Alizé Pharma monitoring standards require full verification for the presence of informed consent, adherence to the inclusion / exclusion criteria, documentation of SAEs and the recording of primary efficacy and safety variables. Additional checks of the consistency of the source data with the eCRFs are performed according to the study-specific monitoring plan. The investigator should provide a designated area where monitoring can occur without interruptions.

The investigator is responsible for completing the eCRFs within 10 days of the patients visit and the Alizé Pharma monitor or designee is responsible for reviewing them, clarifying and resolving any data queries with the involvement of the study team. The completed and corrected eCRFs for completed visits will be electronically sent for data processing. A copy of the eCRFs is retained by the investigator in a CD format, who must ensure that it is stored with other study documents, such as the protocol, the investigators brochure and any protocol amendments, in a secure place.

8.4 Recording of Data and Retention of Documents

8.4.1 Recording of Data

The Sponsor or its designee will instruct the study center regarding data capture procedures on eCRF.

It is the Investigator's responsibility to ensure the accuracy, completeness, and timeliness of the data reported for each patient. Source documentation supporting the data should indicate the patient's participation in the study and should document the dates and details of study procedures, adverse events, and patient status.

The Investigator, or designated representative, should complete data entry as soon as possible after information is collected, preferably on the same day that a study patient is seen for an examination, treatment, or any other study procedure. Any outstanding entries must be completed immediately after the final examination. An explanation should be given for all missing data.

The Investigator must sign and date to endorse the recorded data.

8.4.2 Retention of Documents

The Investigator must maintain confidential all study documentation, and take measures to prevent accidental or premature destruction of these documents.

It is recommended that the Sponsor or designee retain the study documents at least fifteen (15) years after the completion or discontinuation of the Clinical Trial.

However, applicable regulatory requirements should be taken into account in the event that a longer period is required.

If the Sponsor's situation is such that archiving cannot be ensured, Sponsor shall transfer his responsibility for archiving to a mutually agreed designee.

Designee must notify the Sponsor prior to destroying any study essential documents within the fifteen (15) year period following the Clinical Trial completion or discontinuation.

8.5 Auditing Procedures

In addition to the routine monitoring procedures, Alizé Pharma may assign an external auditor to conduct an audit. A regulatory authority may also wish to conduct an inspection during the study or even after its completion. If an inspection is requested by a regulatory authority, the investigator must inform Alizé Pharma or designee immediately that this request has been made.

8.6 Handling of Study Medication

All study medication will be supplied to the study center by the packaging supplier (LC2, Lentilly, France). Drug supplies must be kept in an appropriate, secured area (e.g. locked cabinet) and stored in accordance with the conditions specified on the drug labels. The study center must maintain an accurate record of the shipment and dispensing of the study drug in a drug accountability ledger. An accurate record of the date and amount of study drug dispensed to each subject must be available for inspection at any time. Copies of the drug accountability ledger will be provided to Alizé Pharma or designee by the investigator at the end of the study.

All drug supplies are to be used only for this protocol and not for any other purpose. The investigator must not destroy any drug labels, or any partly-used or unused drug supply. At the conclusion of the study, the investigator will return all used and unused containers, drug labels and a copy of the completed drug disposition form at the address given in the investigator folder provided for the site.

8.7 Insurance

In accordance with the provisions of the law and the GCP, the Sponsor will have an insurance policy intended to guarantee against possible damage resulting from the research.

The studies and/or experiments performed on behalf of the Sponsor will be specifically and expressly guaranteed. It is advisable to underline that noncompliance with the Research Legal Conditions is a cause for guarantee exclusion.

8.8 Publication of Results

Any formal presentation or publication of data collected as a direct or indirect result of this trial will be considered a joint publication by the investigators and the appropriate personnel of Alizé Pharma. It is mandatory that the first publication is based on all data obtained from all analyzed subjects as stipulated in the protocol. Participating investigators must agree not to present data gathered individually or by a subgroup of centers before the full, initial publication. Authorship will be determined according to Alizé Pharma publication policy and in agreement with the coordinating PI. The latter will be the last author and co-authorship will be based on substantial contributions to study conception and design and/or analysis and interpretation of data in accordance with the Vancouver protocol, as well as on recruitment performance when applicable.

Alizé Pharma will manage the process of preparing abstracts, posters and manuscripts for the joint publications that include data from all centers. For any other intended communication on the data from this study, Alizé Pharma requests to receive copies in advance (at least 15 working days for an abstract or oral presentation and 45 working days for a manuscript). This is to allow Alizé Pharma to review the communications for accuracy (thus avoiding potential discrepancies with submissions to regulatory authorities), to verify that confidential information is not being inadvertently divulged, to provide any relevant supplementary information and to allow establishment of co-authorship.

8.9 Disclosure and Confidentiality

By signing the protocol, the investigator agrees to keep all information provided by Alizé Pharma in strict confidence and to request similar confidentiality from his/her staff. Study documents provided by Alizé Pharma (Protocols, investigators' brochure, and other materials) will be stored appropriately to ensure their confidentiality. The information provided by Alizé

Pharma to the investigator may not be disclosed to others without direct written authorization from Alizé Pharma except to the extent necessary to obtain informed consent from patients who wish to participate in the trial.

8.10 Discontinuation of Study

The Sponsor reserves the right to discontinue the study at any time for sufficient reasonable cause. Written notification documenting the reason for study termination will be provided to the investigator. Should the study be closed prematurely, all study materials must be returned to the Sponsor.
